# Effect of empagliflozin on coronary microvascular function in patients with type 2 diabetes mellitus–A randomized, placebo-controlled cross-over study

**DOI:** 10.1371/journal.pone.0263481

**Published:** 2022-02-11

**Authors:** Hannah Elena Suhrs, Malin Nilsson, Kira Bang Bové, Mette Zander, Eva Prescott

**Affiliations:** 1 Department of Cardiology, Bispebjerg Hospital, University of Copenhagen, Copenhagen, Denmark; 2 Department of Endocrinology, Bispebjerg Hospital, University of Copenhagen, Copenhagen, Denmark; Kurume University School of Medicine, JAPAN

## Abstract

**Purpose:**

Results from large scale cardiovascular outcome trials in patients with type 2 diabetes mellitus (DM2) have found that sodium-glucose cotransporter 2 inhibitors (SGLT2i) reduce cardiovascular death and hospitalization for heart failure, but the mechanisms behind the beneficial cardiovascular effects are not fully understood. We tested the hypothesis that the SGLT2i, empagliflozin, improves non-endothelial dependent coronary microvascular function, thereby leading to better cardiac function.

**Methods:**

Patients with DM2 followed at the endocrinology outpatient clinic at Bispebjerg University Hospital were included in a double blinded, placebo-controlled cross-over study. Participants were allocated equally to each treatment sequence using simple randomization and treated with empagliflozin 25 mg and placebo for 12 weeks, interrupted by 2 weeks wash-out period. The primary outcome was coronary microvascular function, assessed as coronary flow velocity reserve (CFVR) and measured with transthoracic doppler echocardiography. Echocardiographic parameters of cardiac function were measured, and blood samples were analyzed for a broad panel of cardiovascular biomarkers.

**Results:**

Thirteen patients were randomized to each sequence and 10 and 9 completed the study according to protocol, respectively, and were included in the analysis of outcome parameters. We found no improvement in CFVR (change in the empagliflozin period was -0.16 (SD 0.58)). There were no effects on cardiac systolic function or indicators of cardiac filling pressure. Well-known effects of empagliflozin were obtained, such as weight loss and reduction in Hba1c level. Creatinine level increased but remained within normal range. We observed a clear trend of reduction in cardiovascular biomarkers after empagliflozin treatment and increased levels after the placebo period. No serious adverse reactions were reported.

**Conclusions:**

Despite effect on weight-loss, Hba1c and biomarkers, treatment with empagliflozin for 12 weeks did not improve CFVR in patients with DM2.

## 1. Introduction

Type 2 diabetes mellitus (DM2) is associated with cardiovascular complications such as atherosclerotic disease, heart failure and coronary microvascular dysfunction (CMD) with impaired vasodilatory reserve [1, 2]. The EMPA-REG outcome study demonstrated that the sodium-glucose cotransporter 2 inhibitor (SGLT2i), empagliflozin, significantly lowered death from cardiovascular causes (38%), heart failure hospitalization (35%) and death from any cause (32%) [3]. The mechanisms behind these beneficial effects remain largely unknown. Several hypotheses have been put forward, and among these it has been suggested that a shift in fuel source from glucose and free fatty acids to the more energy efficient ketogenesis reduces oxidative stress involved in coronary microvascular damage, leading to improved coronary microvascular function [4].

In this study we aimed at evaluating the effect of treatment with empagliflozin on the coronary microvasculature in patients with DM2. We hypothesized that empagliflozin improved coronary microvascular function thereby leading to better cardiac function.

## 2. Methods

### 2.1 Study design

**Fig 1:**
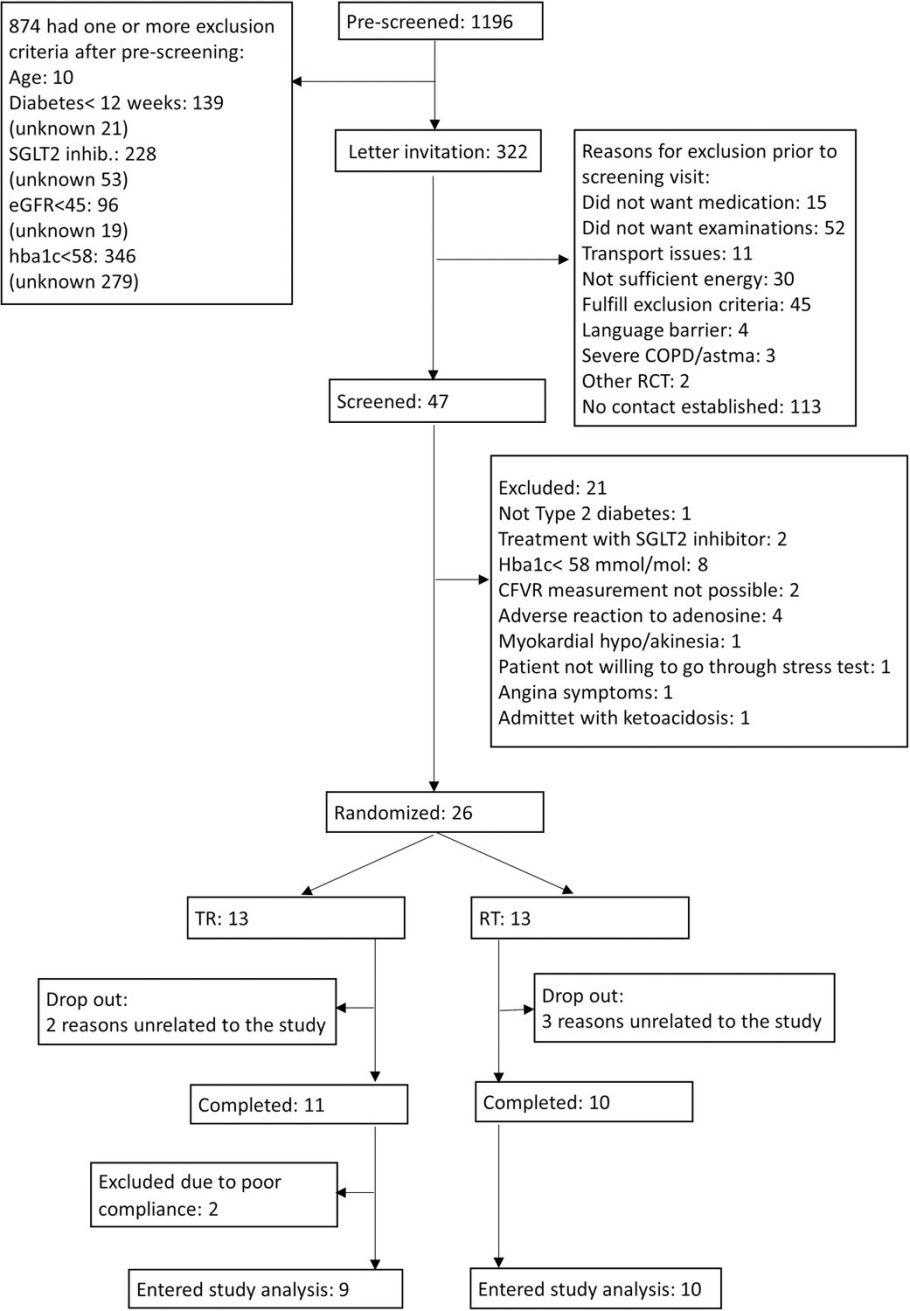
Diagram of participant flow.

**Fig 2:**
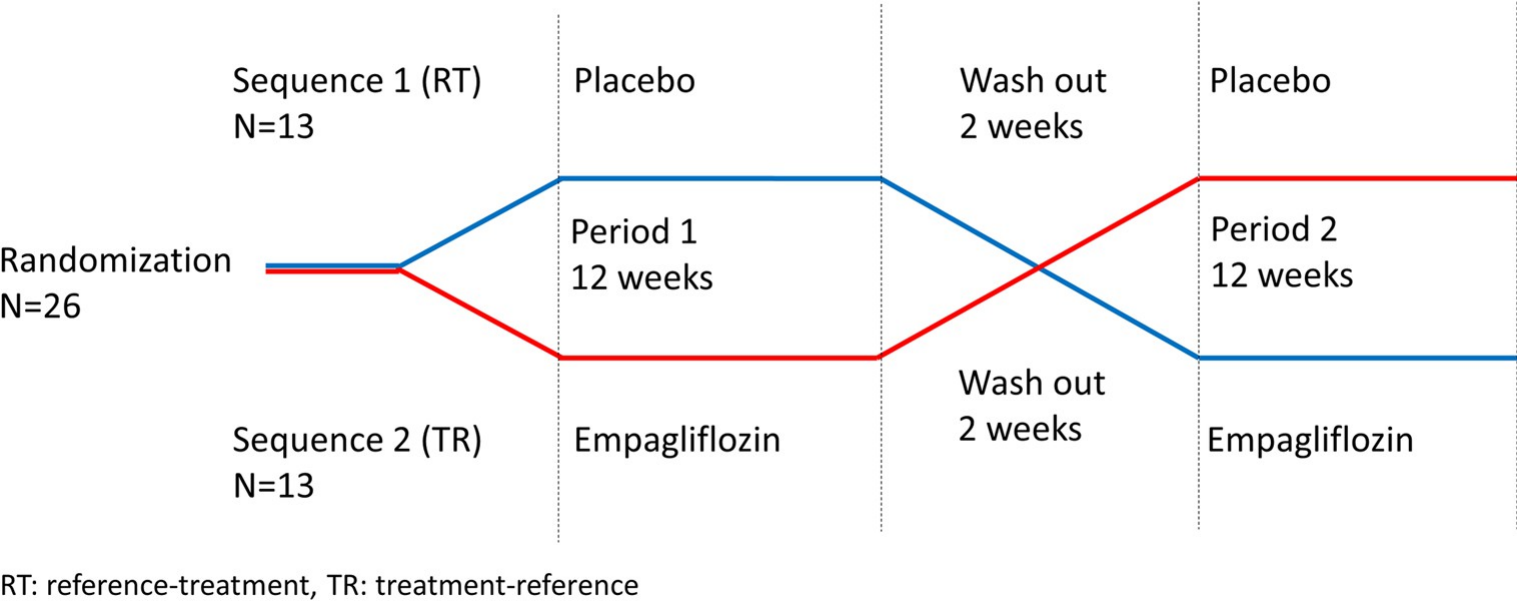
Illustration of cross-over design.

The study design was a randomized, double-blind, placebo controlled cross-over study with a 1:1 allocation ratio. Participant flow chart, Fig 1. Study design, Fig 2.

This design was chosen to eliminate inter subject variance thereby reducing the sample size without losing statistical power. Participants were randomized to either sequence 1: Placebo for 12 weeks followed by a wash out period of 2 weeks and empagliflozin 25 mg for 12 weeks; or sequence 2: empagliflozin 25 mg for 12 weeks followed by a wash-out period of 2 weeks and placebo for 12 weeks. Outcome parameters were evaluated before and after each treatment period.

### 2.2 Study population

Patients followed at the endocrinology outpatient clinic at Bispebjerg University Hospital were pre-screened for inclusion criteria: diagnosis of DM2 for more than 12 weeks, age 40–80 years, no current treatment with an SGLT2i, eGFR≥45 mL/min/1,73m^2^ and HbA1c≥58 mmol/mol. Patients who fulfilled inclusion criteria were invited to a screening visit where inclusion criteria were confirmed, and exclusion criteria evaluated.

### 2.3 Randomization procedure

The study was double-blinded and study medication was prepared by the pharmacy Glostrup Apotek. Medication was delivered in bottles containing placebo tablets or empagliflozin 25 mg, and both bottle containers and tablets were indistinguishable. Id-numbers (1–26) were allocated equally to each treatment sequence using simple randomization and in a consecutive order.

### 2.4 Endpoints

The primary endpoint was change in coronary microvascular function, measured as coronary flow velocity reserve (CFVR) assessed by transthoracic doppler echocardiography (TTDE). Additional secondary endpoints were changes in echocardiographic parameters of cardiac function, change in biochemical measurements and cardiovascular biomarkers.

### 2.5 Examinations

Assessments included clinical and demographic data (age, BMI, hypertension, smoking, and medication). Blood pressure and heart rate measures were obtained after 5 minutes rest. Blood samples were drawn at each visit and analyzed for hemoglobin, HbA1c and creatinine level. Blood ketones were measured in a fasting state with FreeStyre Presicion beta-ketone test strips.

#### 2.5.1 Biomarkers

Blood samples were analyzed by Olink Proteomics, Uppsala, Sweden using the predefined cardiovascular disease panel II and III, measuring 184 protein biomarkers related to the cardiovascular system by real-time polymerase chain reaction. Further description of these biomarker panels is found in previous studies [5, 6] and https://www.olink.com/products/. Studies from our study group on microvascular function have looked for associations with cardiovascular biomarkers using the olink biomarker panels in different populations [5, 6] and therefore we found it interesting to explore possible changes in biomarker levels in this population upon intervention.

#### 2.5.2 Echocardiographic examination

Participants underwent a standard resting transthoracic echocardiography using GE Healthcare Vivid E9 cardiovascular ultrasound system (GE Healthcare, Horten, Norway) with a 1.3–4.0 MHz transducer (GE Vivid 5S probe). Images were stored for off-line analysis (GE EchoPAC v.112, Norway). The same experienced echocardiographer performed all image acquisitions.

*2*.*5*.*2*.*1 Parameters of cardiac systolic function and filling pressure*. We acquired 2-dimensional images of the left ventricle (LV) in apical long axis, 2- and 4-chamber views at frame rates between 60–90 frames/s. Global longitudinal strain (GLS) was measured using software for speckle tracking analysis (Q-analysis, GE EchoPAC v.112, Norway). Aortic valve closure was defined in tissue Doppler M-mode. GLS was calculated as the average of all accepted segmental values of peak systolic strain [7]. Only 3 discarded segments were permitted.

Left ventricular ejection fraction (LVEF) was analyzed as a semi-automated biplane calculation (Auto-EF tool, GE EchoPAC v.112, Norway). Measurements of left ventricle mass index (LVMI) and left atrium volume index (LAI) by the Volume Method of Discs were performed and calculated according to European and American recommendations [8–10]. E/e’ was calculated as a surrogate estimate of left ventricular filling pressures.

*2*.*5*.*2*.*2 Adenosine stress examination*: *CFVR*. Coronary flow velocities (CFV) were measured by TTDE of the left anterior descending artery (LAD) at rest and adenosine infusion (0.14 mg/kg) over 6 minutes using a 2.7–8 MHz transducer (GE Vivid 6S probe) as previously described [11, 12]. The primary endpoint, CFVR, was calculated as the ratio of peak diastolic CFV during adenosine induced hyperemia and rest. Two experts, blinded to participant data, analyzed every CFVR examination independently. The first reading was used, except for estimates that differed by >0.2, in which case the two analyzers reanalyzed the CFVR examination and reached agreement. We have previously reported good inter-analyzer and intra-observer reproducibility of CFVR [13].

Before examinations, participants were instructed to be abstinent from caffeine and food containing significant amount of methylexanthine (coffee, tea, chocolate, cola and banana) and tobacco for 24 hours. Medication containing dipyridamole was paused for 48 hours and anti-hypertensive medication and diuretics for 24 hours.

### 2.6 Compliance

Compliance was assessed by scheduled phone calls and by counting of excess pills in containers returned at the end of each treatment period.

### 2.7 Statistical analysis

Sample size was estimated prior to study commencement for the primary outcome variable: An improvement of 0.23 (i.e. approx. 10%) in CFVR was regarded as clinically relevant. An estimated sample size of 21 was calculated to be necessary for detection of a 0.23 difference in paired means of CFVR with a power of 80% and a two-sided significance level of 5%. Anticipating a 20% dropout rate, enrolment was set at 26 patients. Strict intention-to-treat analysis was not possible due to missing outcome data on participants who dropped out.

Carry over effects were measured using the pkcross command in StataSE 16.1 for cross-over design studies. The command calculates period effects (changes in the variable measured during the course of the trial regardless of intervention); sequence effects (whether the order of interventions affects the result); and carryover effects (whether the effect of an intervention persists in a subsequent period). Using different parameterizations, the treatment effect can be measured in assumption of no carry over, period or sequence effect. Data was analyzed as two-sample *t*-test comparing changes within and between the empagliflozin treatment group and the placebo group after ensuring there was no carry over, sequence or period effect. Paired two-sample *t*-test was used for within allocation comparisons whereas unpaired two-sample *t*-test was used for between treatment allocation comparisons.

Continuous variables are expressed as mean ± SD or as mean (min -max value) where applicable, categorical variables as frequency and percentage. A p-value of ≤ 0.05 was considered statistically significant. All analyses were performed in StataSE 16.1 (Stata Statistical Software: Special edition 16.1 College Station, Texas, USA).

## 3. Results

### 3.1 Population

We screened hospital records of 1,196 patients followed for DM2 at the outpatient clinic at Bispebjerg University Hospital. The most common reasons for exclusion during initial pre-screening were well treated diabetes mellitus (HbA1c<58) and current treatment with an SGLT2i. An invitation was sent to 322 patients and 47 patients were invited to a screening visit after contact by phone. Reasons for exclusion at this stage were principally unwillingness to go through examinations, fulfillment of one or more exclusion criteria or lack of contact. At screening visit patients were interviewed, and a blood sample was taken to confirm in- and exclusion criteria were fulfilled. We included 26 participants between 06-21-2017 and 06-15-2018, and 21 completed the study. Ten participants completed sequence 1: placebo–empagliflozin (RT). Three dropped out because of reasons unrelated to the study, two during the first period and one after the first period (Fig 1).

Eleven participants completed sequence 2: empagliflozin–placebo (TR) with two drop-out because of reasons unrelated to the study during the first period. Two participants were excluded from sequence 2 after having completed both treatment periods after count of excess tablets in the container revealed poor compliance, with the participants taking less than 70% of the planned medication. Therefore 19 participants remained for analysis of outcome parameters.

There was no significant difference between in- and excluded subjects (screened vs. randomized) with regards to age, sex, weight and risk profile including medication and HbA1c.

Mean age was 60 years (min 42, max 73) and a majority of the participants were males. They were obese (mean BMI 30.5 (SD 6.1)) and had a mean HbA1c of 76.26 (SD 16.07) mmol/mol. DM2 was diagnosed approximately 12 years prior to inclusion and cardiovascular disease (CVD) risk factors were common. Participants had normal LVEF and CFVR at baseline was 2.60 (SD 0.56) (Table 1).

**Table 1:** Baseline characteristics.

Variable	sequence = 1 (N = 10)	sequence = 2 (N = 9)	p-value	Total population
**Age (years), mean (min, max)**	59 (42,73)	61 (43, 70)	0.650	60 (42,73)
**Sex (female), n (%)**	2 (20)	5 (56)	0.110	7 (37)
**Weight (kg), mean (SD)**	99.9 (26.0)	89.7 (27.6)	0.420	95.1 (26.5)
**BMI, mean (SD)**	31,0 (5.4)	30.0 (7.1)	0.740	30.5 (6.1)
**Systolic BP (mmHg), mean (SD)**	137.8 (18.2)	135.8 (23.6)	0.840	136.8 (20.4)
**HR, mean (SD)**	74 (12)	73 (5)	0.750	73 (9)
**Smokingstatus**				
**Active, N (%)**	1 (10)	1 (11)	0.760	2 (10)
**Ex-smoker, N (%)**	4 (40)	5 (56)		9 (47)
**Non-smoker, N (%)**	5 (50)	3 (33)		8 (42)
**Years diagnosed with DM2, mean (SD)**	10.4 (10.1)	13.1 (4.8)	0.490	11.6 (8.1)
**Hypertension, n (%)**	7 (70)	4 (50)	0.390	11 (57)
**Dyslipidemia, n (%)**	7 (70)	6 (67)	0.880	15 (68)
**Stroke, n (%)**	1 (10)	1 (13)	0.870	2 (10)
**Insulin treatment, n (%)**	5 (50)	6 (56)	0.810	11 (52)
**GLP1 analogue, n (%)**	1 (10)	3 (33)	0.210	4 (19)
**Oral antidiabetics, n (%)**	10 (100)	8 (89)	0.280	18 (95)
**ACE-inhibitor, n (%)**	4 (40)	1 (11)	0.150	5 (24)
**ARB, n (%)**	1 (10)	0 (0)	0.330	1 (5)
**BB, n (%)**	1 (10)	0 (0)	0.330	1 (5)
**Hgb (mmol/L), mean (SD)**	9.05 (0.86)	8.03 (0.94)	0.024	8.57 (1.01)
**Hba1c (mmol/mol), mean (SD)**	78.70 (19.57)	73.56 (11.59)	0.500	76.26 (16.07)
**Creatinine (umol/L), mean (SD)**	70.3 (13.96)	74.78 (25.08)	0.630	72.42 (19.55)
**LVEF (%), mean (SD)**	57 (5)	57 (5)	0.910	57 (5)
**CFV (m/s), mean (SD)**	0.22 (0.05)	0.24 (0.07)	0.370	0.23 (0.06)
**CFV at hyperemia (m/s), mean (SD)**	0.59 (0.12)	0.57 (0.15)	0.760	0.58 (0.13)
**CFVR, mean (SD)**	2.79 (0.62)	2.39 (0.43)	0.130	2.60 (0.56)

HR: Heart Rate, DM2: diabetes mellitus type 2, GLP1: Glucagon Like Peptide 1, ACE: Angiotensin Converting Enzyme, ARB: Angiotensin-II-receptor-blocker, BB: Beta Blocker, Hgb: Haemoglobin, LVEF: Left Ventricular Ejection Fraction, CFV: Coronary Flow Velocity, CFVR: Coronary Flow Velocity Reserve.

### 3.2 Adherence to treatment

For sequence 1 mean duration of first period was 84 days (min 77, max 91), mean duration of wash-out period was 17 days (min 14, max 22), and mean duration of the second period was 83 days (min 77, max 91). For sequence 2 mean duration of the first period were 79 days (min 77, max 84), mean duration of wash-out period was 21 days (min 14, max 34) and mean duration of the second period was 86 days (min 72 max 91). Mean compliance in the study was 87% (min 74%, max 100%) of time taking the planned medication.

### 3.3 Effect on CFVR

There was no significant effect on the primary outcome, CFVR, after empagliflozin treatment (p = 0.250) nor placebo (p = 0.217) (Table 2).

**Table 2:** Changes in outcome parameters after treatment with empagliflozin and placebo.

Variable	Placebo period, value before (mean, SD)	Placebo period, value after (mean, SD)	P value	Empaglifozin period, value before (mean, SD)	Empagliflozin period, value after (mean, SD)	P value
**Clinical data**						
Weight (kg)	94.3 (26.8)	94.7 (26.3)	0.350	95.0 (26.7)	92.6 (26.0)	<0.001
Systolic BP (mmHg)	134.8 (17.2)	128.3 (14.1)	0.021	131.9 (18.6)	129.6 (18.2)	0.481
Diastolic BP (mmHg)	76.2 (9.5)	72.4 (10.0)	0.016	75.7 (8.8)	74.5 (9.7)	0.461
HR (beat/min)	72 (10)	70 (9)	0.550	71 (8)	69 (9)	0.071
**Echocardiographic measurements**						
CFVR	2.53 (0.63)	2.70 (0.77)	0.217	2.61(0.59)	2.45 (0.75)	0.250
CFV rest (m/s)	0.24 (0.06)	0.25 (0.07)	0.576	0.23 (0.06)	0.23 (0.05)	0.766
CFV hyperaemia (m/s)	0.60 (0.15)	0.65 (0.16)	0.092	0.59 (0.16)	0.55 (0.13)	0.284
LVEF (%)	58 (6)	60 (5)	0.360	57 (5)	58 (5)	0.256
Peak systolic strain (%)	18.2 (3.0)	17.9 (2.7)	0.252	17.5 (2.9)	17.7 (2.6)	0.589
LVMI	82.2 (19.9)	81.1 (20.8)	0.656	77.2 (19.0)	77.6 (18.5)	0.895
LAI	26.0 (5.1)	26.9 (5.1)	0.469	26.3 (5.2)	24.8 (6.3)	0.115
E/e’	10.0 (2.3)	10.4 (3.6)	0.578	9.9 (3.7)	9.5 (2.7)	0.507
**Laboratory data**						
Hba1c (mmol/mol)	70.4 (16.7)	71.3 (11.5)	0.834	72.7 (12.5)	60.0 (11.9)	<0.001
Creatinine (umol/L)	73.1 (19.1)	72.5 (19.3)	0.782	71.6 (19.4)	76.9 (20.6)	<0.001
Ketone rest* (mmol/L)	0.25 (0.20)	0.14 (0.11)	0.012	0.20 (0.17)	0.27 (0.14)	0.073

BP: Blood Pressure, HR: Heart Rate, CFVR: Coronary Flow Velocity Reserve, CFV: Coronary Flow Velocity, LVEF: Left Ventricular Ejection Fraction, LVMI: Left Ventricular Mass Index, LAI: Left Atrial Index.

*significant carry-over effect.

When comparing changes in the two periods the increment in the placebo period and decrement in the empagliflozin period resulted in a significant difference (Table 3). CFV at rest remained unchanged in the two groups. The change was seen during hyperemia where the change in CFV was equal in the two groups but with opposite sign. The difference was small and regarded as a chance finding. There was no carry over effect influencing CFVR measurements.

**Table 3:** Changes in outcome parameters after treatment with empagliflozin compared with placebo.

Variable	Change in placebo period	Change in Empagliflozin period	Difference between changes (95% CI)	P-value
**Clinical data**				
Weight (kg)	0.39 (1.79)	-2.45 (2.49)	2.84 (1.47, 4.21)	<0.001
Systolic BP (mmHg)	-6.42 (11.01)	-2.32 (14.02)	-4.11 (-11.51, 3.30)	0.260
Diastolic BP (mmHg)	-3.8 (6.18)	-1.2 (7.00)	-2.6 (-6.32, 1.16)	0.164
HR (beats/min)	-0.27 (8.84)	-2.11 (4.23)	0.84 (-3.82, 5.49)	0.709
**Echocardiographic measurements**				
CFVR	0.18 (0.60)	-0.16 (0.58)	0.33 (0.23, 0.64)	0.037
CFV rest	0.01 (0.06)	0.00 (0.05)	0.00 (-0.03, 0.04)	0.778
CFV hyperaemia	0.05 (0.13)	-0.04 (0.15)	0.09 (0.00, 0.18)	0.044
LVEF (%)	1.2 (5.8)	1.5 (5.6)	-0.2 (-4.85, 4.36)	0.911
Peak systolic strain (%)	-0.3 (1.3)	0.2 (1.7)	-0.6 (-1.56, 0.45)	0.259
LVMI	-1.0 (9.6)	0.4 (13.5)	-1.4 (-11.41, 8.57)	0.769
LAI	0.9 (5.2)	-1.5 (4.0)	2.4 (-0.03, 4.82)	0.052
E/e’	0.4 (3.3)	-0.4 (2.4)	0.8 (-1.32, 2.93)	0.439
**Laboratory data**				
Hba1c (mmol/mol)	0.8 (17.3)	-12.7 (10.4)	13.5 (2.57, 24.48)	0.018
Creatinine (umol/L)	-0.6 (9.8)	5.3 (5.5)	-5.9 (-11.43, -0.36)	0.038
Ketone rest* (mmol/L)	-0.11 (0.18)	0.07 (0.16)	-0.18 (-0.30, -0.06)	0.005

HR: Heart Rate, CFVR: Coronary Flow Velocity Reserve, CFV: Coronary Flow Velocity, LVEF: Left Ventricular Ejection Fraction, LVMI: Left Ventricular Mass Index, LAI: Left Atrial Index

*significant carry-over effect.

### 3.4 Additional outcomes

Empagliflozin treatment resulted in a significant weight loss (p<0.001) and, concomitantly, a significant reduction in HbA1c (p<0.001) whereas creatinine increased significantly but remained within the normal range (Table 2). The changes remained significant when compared with the placebo period (Table 3).

Ketone levels increased after empagliflozin treatment, but the increment did not reach statistical significance (0.073) and the measurements were influenced by carry-over effect. No effect was seen on blood pressure, LVEF or peak systolic strain.

**Fig 3:**
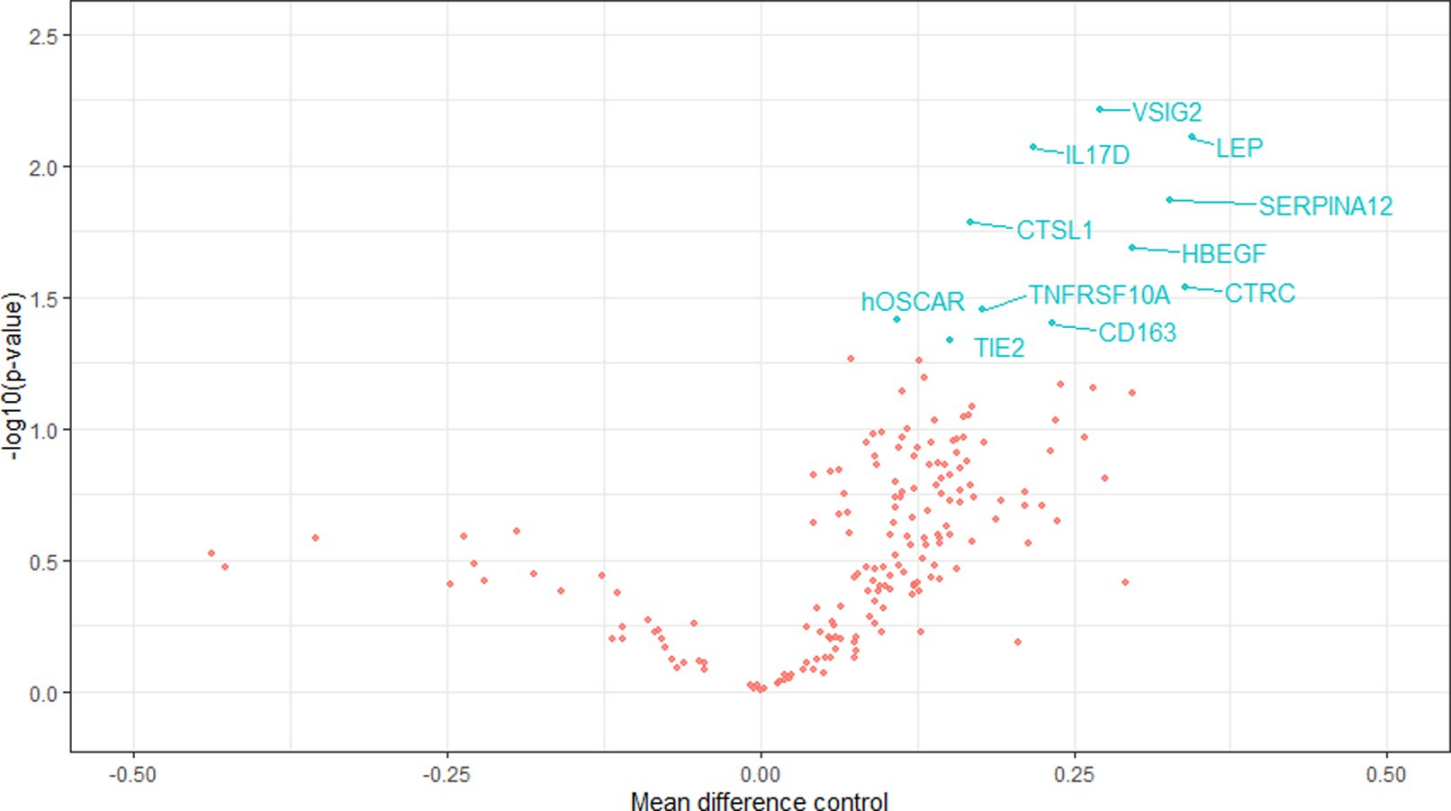
The figure gives a Volcano plot depicting negative logarithm of p-value (y) against regression coefficient (x) for each of the 184 biomarkers in the placebo period. Biomarkers that changed significantly are noted with blue text. Reduction in biomarker-level shown on the left, increase in biomarker level on the right.

**Fig 4:**
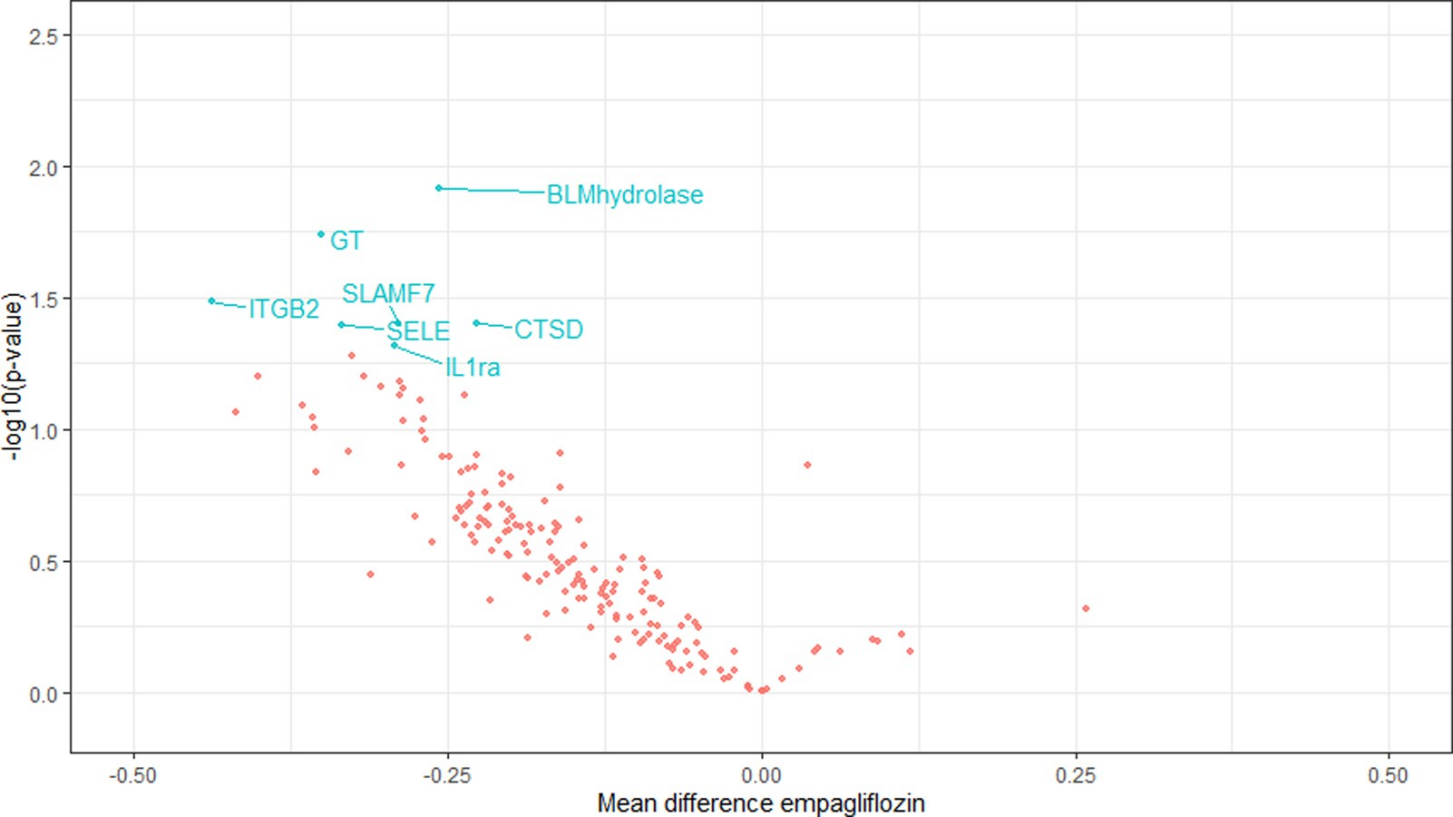
The figure gives a Volcano plot depicting negative logarithm of p-value (y) against regression coefficient (x) for each of the 184 biomarkers in the empagliflozin period. Biomarkers that changed significantly are noted with blue text. Reduction in biomarker-level shown on the left, increase in biomarker level on the right.

There was a clear trend of reduction in cardiovascular biomarkers after empagliflozin treatment and increased levels after the placebo period. Fig 3 gives in blue the biomarkers with significant increase during placebo treatment and Fig 4 gives in blue the biomarkers with significant decrease during empagliflozin treatment.

See https://www.olink.com/resources-support/document-download-center/ for a table of the biomarkers measured (Olink cardiovascular II and cardiovascular III) and S1 Table for the effect of placebo versus empagliflozin.

### 3.5 Adverse events

Empagliflozin was generally well-tolerated. During active treatment 6 adverse events and 4 adverse reactions were registered but no participant experienced a serious adverse event or reaction. Adverse reactions experienced during active treatment were vaginal candidiasis, balanitis and skin infection, one had an incidence of sensation of hypoglycemia, but no blood glucose was measured. Four adverse events were experienced during placebo treatment. None of the adverse reactions resulted in dropout of the study.

## 4. Discussion

In the present study we did not find evidence of improvement of coronary microcirculatory function after treatment with empagliflozin. We did see a significant loss of body weight and a decrease in HbA1c after treatment with empagliflozin that remained significant when compared with the placebo group. Blood ketones increased after 12 weeks of treatment with empagliflozin, but the measurement was influenced by carry-over effect and did not reach statistical significance.

Previous studies have demonstrated that SGLT2i promote a shift to fasting state metabolism characterized by reduced blood glucose and increased lipid oxidation leading to an increase in blood ketone levels [14]. It is well established that under conditions of diabetes mellitus and/or heart failure the metabolic flexibility of the heart is impaired. It has been speculated that under these circumstances, availability of ketones as an alternative and more efficient energy source may explain some of the beneficial cardiac effects seen with SGLT2i treatment.

A previous study found that ketone bodies displace myocardial glucose uptake and increase myocardial blood flow in healthy humans (measured with PET scans), indicating that ketone bodies are important cardiac fuels and vasodilators [4]. Furthermore a recent study has found that acute 3-hydroxybutyrate infusion reduced cerebral glucose uptake and increased cerebral blood flow in the brain, measured by PET scans [15]. Another recent cross-over study in 13 patients with DM2 found no effect of empagliflozin on myocardial free fatty acid uptake measured with 11C-palmitate and 18F-fluorodeoxyglucose PET/CT, but myocardial substrate utilization shifted from glucose toward other sources, and resting myocardial blood flow was reduced [16].

However, the increased ketone levels in the present study did not affect coronary microvascular function, measured as CFVR.

Preclinical data have suggested that empagliflozin reduces arterial stiffness [17] and improves coronary microvascular function (measured noninvasively as CFVR by Doppler ultrasound imaging) and contractile performance alongside with metabolic changes in a mice model for diabetes mellitus and heart failure [18].

Involvement of the endothelium has also been explored in another preclinical study demonstrating that cardiac microvascular endothelial cells improve cardiomyocyte contraction and relaxation in a co-culture model of cardiac microvascular endothelial cells and cardiomyocytes isolated from adult rats, an effect that was lost after pre-incubation of cardiac microvascular endothelial cells with the inflammatory mediator TNF-a. Evidence was provided that empagliflozin restored this beneficial effect of cardiac microvascular endothelial cells by reducing mitochondrial reactive oxygen species (ROS) production and cytoplasmic ROS accumulation, which led to restoration of endothelial nitric oxide (NO) bioavailability and preservation of cardiomyocyte contraction and relaxation [19].

Inflammation has been associated with CMD and cardiac diastolic dysfunction, conditions that are common in patients with diabetes mellitus and are linked to heart failure with preserved ejection fraction [20–22]. A preclinical trial has reported that empagliflozin improved cardiac diastolic function by increasing cGMP-dependent titin phosphorylation in human ventricular trabeculae and in a murine model of heart failure with preserved ejection fraction [23] and another preclinical trial showed that empagliflozin acts directly on sodium and calcium exchange in isolated cardiomyocytes [24].

Even though diabetes mellitus is associated with microvascular dysfunction and previous studies have documented that CMD is common in this population [2, 25, 26], baseline CFVR in our population was 2.60 which is above cut-off level for CMD used in most prognostic studies [27]. Sub analysis in patients with CFVR<2.5 and 2.0 were not performed due to the small study size. Furthermore our study population had normal LVEF and we know from the EMPA-REG outcome study [3] and the DAPA-HF study [28] that most benefit of SGLT2i treatment is seen in patients with heart failure. Thus, is it possible that an effect of empagliflozin on coronary microvascular function would be seen in patients with severe CMD and/or heart failure.

In line with our results a recently published randomized study of 90 patients with DM2 and known cardiovascular disease or high cardiovascular disease risk found no effect of empagliflozin for 13 weeks on myocardial flow reserve (MFR) measured by 82Rb-PET/CT. Mean MFR was 2.2 at baseline in their study, which is only moderately reduced considering the high-risk population and may also explain the lack of treatment response [29].

We analyzed blood samples for a large number of cardiovascular biomarkers and found a clear trend of reduction in cardiovascular biomarkers after empagliflozin treatment and increased levels after the placebo period. Due to the large number of biomarkers studied relative to the small number of study participants there was a risk of type 1 error and the analysis can only be regarded as exploratory.

In future studies it would be interesting to explore if treatment with empagliflozin is associated with a decrease in cardiovascular inflammatory biomarkers.

### 4.1 Strengths and limitations

We experienced generally good adherence to study protocol. Participants were examined by the same doctors throughout the study period.

Unfortunately, we did not have sufficient power to detect a mean change in CFVR of 0.23 ie 10%. With inclusion of 19 subjects we obtained a power of 0.77. The standard deviation of the mean change in the placebo period and the empagliflozin period was approximately 0.60, which was larger than expected and allows detection of a change in CFVR of 0.65 with a power of 77%. Thus, we might have overseen a small effect of empagliflozin on the microvasculature, however, the observed changes were in the opposite direction of the hypothesis.

Participants were not evaluated for macrovascular coronary artery disease prior to inclusion in the study. However, none of the participants were previously revascularized or had known CVD. None of the participants described symptoms of angina pectoris. To reduce probability of macrovascular coronary artery disease, participants were evaluated for signs of regional hypokinesia at stress echocardiography. However, we cannot rule out the possibility that macrovascular disease of the LAD may have influenced measurement of coronary flow reserve.

We measured non-endothelial dependent coronary vascular function and therefore we cannot rule out a possible effect of empagliflozin on endothelial function of the coronary microvessels.

This was a single center study and a high proportion of the participants were males of caucasian ethnicity and therefore the results may not extend to different populations.

## 5. Conclusion

Despite effect on weight-loss, Hba1c and biomarkers, empagliflozin treatment for 12 weeks did not improve CFVR in patients DM2. This study does not support that non-endothelial dependent coronary microvascular function is involved in the beneficial effect of SGLT2i.

## 6. Ethics approval and consent to participate

The study was approved by The Danish Research Ethics Committee (H-17004197), The Danish Health and Medicines Authorities (EudraCT 2017-000240-17), registered at the EU Clinical Trials Register on the 2^nd^ of May 2017, https://www.clinicaltrialsregister.eu/ctr-search/search?query=2017-000240-17, and has been continuously monitored by the GCP-unit at Bispebjerg University Hospital, Denmark. All participants gave written informed consent on oral and written information.

The full protocol can be accessed by contacting Hannah Elena Suhrs, hannah.elena.suhrs@regionh.dk

## Supporting information

S1 Table Effect of placebo versus empagliflozin on 184 cardiovascular biomarkers. (DOCX)

S1 File (PDF)

S2 File (DOCX)
