# Supplementary material for: Effect of empagliflozin on coronary microvascular function in patients with type 2 diabetes mellitus–A randomized, placebo-controlled cross-over study
Source: PLoS One. 2022 Feb 11;17(2):e0263481. doi: 10.1371/journal.pone.0263481 (PMC8836314; doi:10.1371/journal.pone.0263481)
Supplement: S1 Table — (DOCX) [file pone.0263481.s001.docx]

| **Effect of placebo versus empagliflozin on 184 cardiovascular biomarkers** | | | | | | | | | |
| --- | --- | --- | --- | --- | --- | --- | --- | --- | --- |
| **Variable name** | **Before placebo** | **After placebo** | **p** | **Before empagliflozin** | **After empagliflozin** | **p** | **Difference in placebo period** | **Difference in empagliflozin period** | **p** |
| **ACE2** | 4,525 | 4,694 | 0,083 | 4,610 | 4,294 | 0,063 | 0,169 | -0,316 | **0,034** |
| **ADAMTS13** | 7,302 | 7,344 | 0,228 | 7,261 | 7,240 | 0,701 | 0,042 | -0,021 | 0,256 |
| **ADM** | 7,666 | 7,872 | 0,653 | 8,214 | 8,029 | 0,621 | 0,206 | -0,185 | 0,581 |
| **AGRP** | 5,357 | 5,517 | 0,141 | 5,484 | 5,416 | 0,665 | 0,160 | -0,068 | 0,306 |
| **ALCAM** | 7,548 | 7,659 | 0,333 | 7,734 | 7,448 | 0,138 | 0,111 | -0,286 | 0,063 |
| **AMBP** | 8,609 | 8,682 | **0,054** | 8,625 | 8,576 | 0,570 | 0,072 | -0,050 | 0,192 |
| **ANGPT1** | 11,617 | 11,708 | 0,128 | 11,618 | 11,555 | 0,559 | 0,092 | -0,063 | 0,192 |
| **APN** | 5,811 | 5,903 | 0,342 | 5,986 | 5,754 | 0,191 | 0,092 | -0,232 | 0,110 |
| **AXL** | 8,721 | 8,850 | 0,311 | 8,881 | 8,680 | 0,305 | 0,130 | -0,201 | 0,155 |
| **AZU1** | 6,274 | 6,047 | 0,330 | 6,344 | 6,318 | 0,880 | -0,227 | -0,025 | 0,480 |
| **BLMhydrolase** | 3,204 | 3,261 | 0,549 | 3,377 | 3,121 | **0,012** | 0,057 | -0,256 | **0,036** |
| **BMP6** | 4,932 | 4,933 | 0,998 | 5,124 | 4,770 | 0,146 | 0,001 | -0,355 | 0,432 |
| **BNP** | 1,936 | 2,040 | 0,255 | 1,978 | 1,925 | 0,541 | 0,104 | -0,052 | 0,215 |
| **BOC** | 4,547 | 4,595 | 0,592 | 4,607 | 4,407 | 0,152 | 0,048 | -0,199 | 0,187 |
| **CA5A** | 3,578 | 3,746 | 0,269 | 3,513 | 3,304 | 0,268 | 0,169 | -0,209 | 0,175 |
| **CASP3** | 6,727 | 6,301 | 0,335 | 6,687 | 6,377 | 0,356 | -0,427 | -0,310 | 0,827 |
| **CCL15** | 8,078 | 8,221 | 0,273 | 8,238 | 8,240 | 0,993 | 0,143 | 0,001 | 0,514 |
| **CCL16** | 7,245 | 7,301 | 0,750 | 7,367 | 7,277 | 0,605 | 0,056 | -0,090 | 0,521 |
| **CCL17** | 11,213 | 11,278 | 0,635 | 11,284 | 11,107 | 0,383 | 0,065 | -0,177 | 0,409 |
| **CCL24** | 6,297 | 6,311 | 0,935 | 6,402 | 6,257 | 0,357 | 0,014 | -0,145 | 0,491 |
| **CCL3** | 8,291 | 8,212 | 0,637 | 8,237 | 7,975 | 0,268 | -0,079 | -0,261 | 0,581 |
| **CD163** | 8,445 | 8,677 | **0,040** | 8,700 | 8,335 | 0,082 | 0,232 | -0,365 | **0,013** |
| **CD4** | 5,527 | 5,620 | 0,138 | 5,574 | 5,452 | 0,386 | 0,093 | -0,123 | 0,186 |
| **CD40L** | 9,130 | 9,122 | 0,955 | 9,211 | 9,014 | 0,217 | -0,008 | -0,197 | 0,449 |
| **CD84** | 6,746 | 6,792 | 0,483 | 6,781 | 6,632 | 0,312 | 0,045 | -0,149 | 0,284 |
| **CD93** | 11,008 | 11,116 | 0,201 | 11,123 | 10,882 | 0,199 | 0,108 | -0,240 | 0,085 |
| **CDH5** | 3,647 | 3,791 | 0,261 | 3,844 | 3,557 | 0,074 | 0,143 | -0,288 | **0,048** |
| **CEACAM8** | 7,566 | 7,387 | 0,360 | 7,523 | 7,430 | 0,635 | -0,180 | -0,093 | 0,797 |
| **CHI3L1** | 6,339 | 6,466 | 0,415 | 6,642 | 6,285 | 0,090 | 0,127 | -0,357 | **0,035** |
| **CHIT1** | 4,216 | 4,275 | 0,562 | 4,322 | 4,133 | 0,273 | 0,059 | -0,189 | 0,182 |
| **CNTN1** | 4,943 | 5,037 | 0,414 | 5,143 | 4,858 | 0,093 | 0,094 | -0,285 | 0,079 |
| **COL1A1** | 2,754 | 2,840 | 0,418 | 2,807 | 2,647 | 0,168 | 0,086 | -0,160 | 0,152 |
| **CPA1** | 6,010 | 6,285 | 0,156 | 6,316 | 6,100 | 0,449 | 0,275 | -0,216 | 0,113 |
| **CPB1** | 6,278 | 6,575 | 0,073 | 6,641 | 6,471 | 0,506 | 0,297 | -0,170 | 0,113 |
| **CSTB** | 4,972 | 5,024 | 0,745 | 5,226 | 5,001 | 0,236 | 0,052 | -0,225 | 0,220 |
| **CTRC** | 10,864 | 11,203 | **0,029** | 10,939 | 10,866 | 0,775 | 0,339 | -0,073 | 0,181 |
| **CTSD** | 2,478 | 2,550 | 0,250 | 2,623 | 2,397 | **0,039** | 0,072 | -0,226 | **0,014** |
| **CTSL1** | 8,346 | 8,515 | **0,016** | 8,417 | 8,244 | 0,188 | 0,168 | -0,173 | **0,049** |
| **CTSZ** | 5,124 | 5,261 | 0,113 | 5,313 | 5,076 | 0,074 | 0,137 | -0,236 | **0,029** |
| **CXCL1** | 11,573 | 11,681 | 0,305 | 11,571 | 11,428 | 0,382 | 0,108 | -0,143 | 0,260 |
| **CXCL16** | 6,139 | 6,306 | 0,164 | 6,305 | 6,134 | 0,359 | 0,168 | -0,171 | 0,097 |
| **DCN** | 5,374 | 5,488 | 0,072 | 5,418 | 5,330 | 0,441 | 0,113 | -0,088 | 0,122 |
| **DECR1** | 5,039 | 4,881 | 0,415 | 4,982 | 4,902 | 0,638 | -0,158 | -0,081 | 0,709 |
| **DLK1** | 6,242 | 6,357 | 0,351 | 6,424 | 6,194 | 0,255 | 0,115 | -0,230 | 0,113 |
| **Dkk1** | 11,330 | 11,458 | 0,056 | 11,349 | 11,233 | 0,394 | 0,128 | -0,117 | 0,131 |
| **EGFR** | 2,736 | 2,801 | 0,476 | 2,845 | 2,700 | 0,222 | 0,065 | -0,145 | 0,143 |
| **EPHB4** | 5,600 | 5,763 | 0,108 | 5,765 | 5,573 | 0,234 | 0,163 | -0,192 | 0,063 |
| **EpCAM** | 5,662 | 5,821 | 0,192 | 5,781 | 5,564 | 0,231 | 0,159 | -0,216 | 0,081 |
| **FABP2** | 9,578 | 9,494 | 0,594 | 9,450 | 9,421 | 0,898 | -0,084 | -0,029 | 0,849 |
| **FABP4** | 5,505 | 5,717 | 0,196 | 5,652 | 5,641 | 0,966 | 0,212 | -0,011 | 0,377 |
| **FAS** | 6,334 | 6,456 | 0,395 | 6,494 | 6,332 | 0,346 | 0,122 | -0,161 | 0,225 |
| **FGF21** | 7,865 | 8,157 | 0,389 | 8,411 | 7,693 | **0,020** | 0,292 | -0,718 | **0,044** |
| **FGF23** | 2,829 | 2,971 | 0,135 | 3,167 | 3,040 | 0,495 | 0,142 | -0,127 | 0,260 |
| **FS** | 11,869 | 11,930 | 0,620 | 11,794 | 11,795 | 0,997 | 0,061 | 0,001 | 0,830 |
| **GDF15** | 5,983 | 6,220 | 0,225 | 6,227 | 6,170 | 0,796 | 0,237 | -0,057 | 0,273 |
| **GDF2** | 9,765 | 9,897 | 0,276 | 9,883 | 9,466 | 0,087 | 0,132 | -0,418 | 0,056 |
| **GH** | 9,559 | 9,494 | 0,819 | 9,321 | 9,203 | 0,740 | -0,066 | -0,118 | 0,918 |
| **GIF** | 8,067 | 8,143 | 0,649 | 8,121 | 7,833 | 0,066 | 0,076 | -0,288 | 0,193 |
| **GLO1** | 7,327 | 7,586 | 0,108 | 8,104 | 7,167 | **0,005** | 0,259 | -0,937 | **0,006** |
| **GP6** | 3,959 | 3,870 | 0,540 | 4,106 | 3,889 | 0,196 | -0,089 | -0,217 | 0,601 |
| **GRN** | 5,451 | 5,599 | 0,137 | 5,624 | 5,385 | 0,147 | 0,148 | -0,238 | **0,047** |
| **GT** | 3,619 | 3,509 | 0,568 | 3,610 | 3,259 | **0,018** | -0,110 | -0,350 | 0,365 |
| **Gal3** | 5,112 | 5,167 | 0,621 | 5,292 | 5,164 | 0,425 | 0,055 | -0,127 | 0,322 |
| **Gal4** | 5,000 | 5,077 | 0,623 | 5,102 | 4,939 | 0,322 | 0,077 | -0,163 | 0,264 |
| **Gal9** | 8,884 | 8,997 | 0,108 | 8,936 | 8,842 | 0,417 | 0,113 | -0,094 | 0,129 |
| **HAOX1** | 7,354 | 7,244 | 0,631 | 7,167 | 6,470 | **0,046** | -0,110 | -0,698 | 0,212 |
| **HBEGF** | 6,911 | 7,208 | **0,021** | 7,063 | 6,861 | 0,298 | 0,296 | -0,202 | **0,040** |
| **HO1** | 11,714 | 11,802 | 0,524 | 11,930 | 11,654 | 0,214 | 0,088 | -0,276 | 0,242 |
| **HSP27** | 9,380 | 9,440 | 0,696 | 9,743 | 9,156 | **0,033** | 0,060 | -0,587 | 0,065 |
| **ICAM2** | 5,672 | 5,816 | 0,156 | 5,858 | 5,587 | 0,077 | 0,144 | -0,271 | **0,033** |
| **IDUA** | 6,856 | 6,775 | 0,589 | 6,928 | 6,675 | 0,128 | -0,081 | -0,253 | 0,488 |
| **IGFBP1** | 6,633 | 6,386 | 0,389 | 6,509 | 6,768 | 0,485 | -0,247 | 0,259 | 0,242 |
| **IGFBP2** | 8,470 | 8,595 | 0,384 | 8,631 | 8,495 | 0,569 | 0,126 | -0,136 | 0,277 |
| **IGFBP7** | 8,825 | 9,013 | 0,221 | 9,003 | 8,817 | 0,368 | 0,188 | -0,186 | 0,145 |
| **IL16** | 7,245 | 7,394 | 0,235 | 7,439 | 7,253 | 0,362 | 0,149 | -0,187 | 0,215 |
| **IL17D** | 2,831 | 3,049 | **0,009** | 2,959 | 2,873 | 0,444 | 0,217 | -0,086 | **0,049** |
| **IL17RA** | 4,680 | 4,631 | 0,765 | 4,742 | 4,596 | 0,372 | -0,049 | -0,147 | 0,623 |
| **IL18** | 9,449 | 9,615 | 0,089 | 9,582 | 9,313 | 0,102 | 0,167 | -0,270 | **0,047** |
| **IL18BP** | 6,576 | 6,697 | 0,220 | 6,707 | 6,525 | 0,248 | 0,121 | -0,183 | 0,094 |
| **IL1RL2** | 5,136 | 5,271 | 0,138 | 5,236 | 5,005 | 0,177 | 0,135 | -0,231 | 0,063 |
| **IL1RT1** | 6,142 | 6,293 | 0,151 | 6,362 | 6,059 | 0,069 | 0,151 | -0,303 | **0,029** |
| **IL1RT2** | 6,149 | 6,307 | 0,125 | 6,376 | 6,051 | **0,053** | 0,157 | -0,325 | **0,015** |
| **IL1ra** | 6,417 | 6,515 | 0,484 | 6,498 | 6,206 | **0,049** | 0,099 | -0,291 | 0,109 |
| **IL27** | 6,823 | 6,934 | 0,182 | 6,856 | 6,805 | 0,650 | 0,111 | -0,051 | 0,285 |
| **IL2RA** | 4,460 | 4,598 | 0,094 | 4,653 | 4,425 | 0,140 | 0,139 | -0,228 | **0,040** |
| **IL4RA** | 3,040 | 3,171 | 0,064 | 3,106 | 3,014 | 0,383 | 0,131 | -0,092 | 0,097 |
| **IL6** | 4,433 | 4,646 | 0,275 | 4,636 | 4,368 | 0,092 | 0,213 | -0,268 | 0,116 |
| **IL6RA** | 12,383 | 12,528 | 0,177 | 12,533 | 12,388 | 0,442 | 0,145 | -0,145 | 0,115 |
| **ITGB1BP2** | 2,714 | 2,661 | 0,551 | 2,668 | 2,461 | 0,149 | -0,053 | -0,206 | 0,374 |
| **ITGB2** | 6,111 | 6,135 | 0,890 | 6,419 | 5,981 | **0,033** | 0,024 | -0,438 | 0,079 |
| **IgGFcreceptorIIb** | 4,208 | 4,315 | 0,228 | 4,252 | 4,088 | 0,228 | 0,107 | -0,164 | 0,153 |
| **JAMA** | 7,257 | 6,819 | 0,300 | 6,819 | 6,937 | 0,702 | -0,438 | 0,118 | 0,289 |
| **KIM1** | 10,295 | 10,429 | 0,206 | 10,455 | 10,055 | 0,063 | 0,134 | -0,400 | **0,043** |
| **KLK6** | 4,672 | 4,795 | 0,127 | 4,787 | 4,730 | 0,521 | 0,123 | -0,058 | 0,102 |
| **LDLreceptor** | 6,093 | 6,189 | 0,595 | 6,169 | 5,840 | 0,123 | 0,097 | -0,329 | 0,105 |
| **LEP** | 7,227 | 7,571 | **0,008** | 7,385 | 7,149 | 0,233 | 0,344 | -0,236 | **0,020** |
| **LOX1** | 10,282 | 10,156 | 0,362 | 10,230 | 10,293 | 0,705 | -0,126 | 0,063 | 0,460 |
| **LPL** | 9,854 | 9,975 | 0,429 | 10,011 | 9,796 | 0,291 | 0,121 | -0,215 | 0,253 |
| **LTBR** | 4,137 | 4,241 | 0,413 | 4,235 | 4,131 | 0,521 | 0,104 | -0,104 | 0,277 |
| **MARCO** | 7,543 | 7,627 | 0,113 | 7,560 | 7,477 | 0,352 | 0,085 | -0,083 | 0,064 |
| **MB** | 6,933 | 6,958 | 0,866 | 7,144 | 7,074 | 0,694 | 0,024 | -0,070 | 0,705 |
| **MCP1** | 5,755 | 5,891 | 0,369 | 5,941 | 5,767 | 0,238 | 0,136 | -0,175 | 0,208 |
| **MEPE** | 4,261 | 4,335 | 0,741 | 4,447 | 4,331 | 0,532 | 0,075 | -0,116 | 0,547 |
| **MERTK** | 6,647 | 6,826 | 0,113 | 6,847 | 6,492 | 0,099 | 0,179 | -0,355 | 0,058 |
| **MMP12** | 8,380 | 8,470 | 0,380 | 8,472 | 8,247 | 0,219 | 0,090 | -0,224 | 0,179 |
| **MMP2** | 3,152 | 3,363 | 0,175 | 3,348 | 3,207 | 0,439 | 0,211 | -0,141 | 0,130 |
| **MMP3** | 6,858 | 7,082 | 0,196 | 7,063 | 6,976 | 0,551 | 0,224 | -0,087 | 0,197 |
| **MMP7** | 12,925 | 13,022 | 0,104 | 12,962 | 12,845 | 0,417 | 0,097 | -0,117 | 0,210 |
| **MMP9** | 7,849 | 7,805 | 0,779 | 7,927 | 7,884 | 0,730 | -0,044 | -0,044 | 0,999 |
| **MPO** | 5,034 | 4,921 | 0,426 | 5,116 | 5,004 | 0,341 | -0,114 | -0,112 | 0,993 |
| **NEMO** | 5,343 | 5,268 | 0,682 | 5,713 | 5,159 | **0,034** | -0,075 | -0,554 | 0,156 |
| **NTproBNP** | 2,685 | 2,813 | 0,600 | 2,984 | 2,745 | 0,205 | 0,128 | -0,239 | 0,172 |
| **Notch3** | 4,996 | 5,139 | 0,378 | 5,148 | 4,929 | 0,225 | 0,143 | -0,219 | 0,138 |
| **OPG** | 4,086 | 4,128 | 0,827 | 4,052 | 3,992 | 0,708 | 0,043 | -0,060 | 0,663 |
| **OPN** | 7,611 | 7,688 | 0,698 | 7,764 | 7,651 | 0,629 | 0,077 | -0,113 | 0,535 |
| **PAI** | 8,750 | 8,873 | 0,397 | 8,891 | 8,764 | 0,407 | 0,123 | -0,126 | 0,250 |
| **PAPPA** | 4,016 | 4,168 | 0,188 | 4,135 | 3,951 | 0,232 | 0,152 | -0,185 | 0,109 |
| **PAR1** | 6,283 | 6,321 | 0,567 | 6,313 | 6,172 | 0,278 | 0,038 | -0,141 | 0,274 |
| **PARP1** | 2,328 | 2,445 | 0,101 | 2,391 | 2,408 | 0,897 | 0,117 | 0,017 | 0,431 |
| **PCSK9** | 3,468 | 3,560 | 0,557 | 3,534 | 3,382 | 0,324 | 0,092 | -0,152 | 0,285 |
| **PDGFsubunitA** | 5,767 | 5,919 | 0,253 | 5,894 | 5,779 | 0,516 | 0,152 | -0,115 | 0,223 |
| **PDGFsubunitB** | 12,007 | 12,070 | 0,212 | 11,964 | 11,884 | 0,463 | 0,063 | -0,080 | 0,213 |
| **PDL2** | 4,314 | 4,476 | 0,091 | 4,341 | 4,178 | 0,247 | 0,162 | -0,164 | 0,109 |
| **PECAM1** | 5,849 | 5,495 | 0,264 | 5,503 | 5,494 | 0,970 | -0,355 | -0,010 | 0,403 |
| **PGF** | 8,192 | 8,432 | 0,068 | 8,250 | 8,048 | 0,246 | 0,240 | -0,203 | 0,067 |
| **PGLYRP1** | 8,746 | 8,761 | 0,923 | 8,852 | 8,898 | 0,686 | 0,015 | 0,046 | 0,874 |
| **PI3** | 2,606 | 2,609 | 0,973 | 2,615 | 2,658 | 0,705 | 0,004 | 0,042 | 0,793 |
| **PIgR** | 5,814 | 5,812 | 0,945 | 5,816 | 5,853 | 0,137 | -0,002 | 0,037 | 0,267 |
| **PLC** | 8,688 | 8,618 | 0,756 | 8,571 | 8,664 | 0,645 | -0,070 | 0,093 | 0,564 |
| **PON3** | 5,184 | 5,341 | 0,343 | 5,391 | 5,295 | 0,653 | 0,157 | -0,096 | 0,394 |
| **PRELP** | 9,080 | 9,150 | 0,209 | 9,165 | 9,055 | 0,307 | 0,070 | -0,110 | 0,183 |
| **PRSS27** | 10,304 | 10,411 | 0,182 | 10,359 | 10,132 | 0,126 | 0,107 | -0,227 | 0,087 |
| **PRSS8** | 9,991 | 10,102 | 0,119 | 9,985 | 9,779 | 0,162 | 0,111 | -0,206 | **0,048** |
| **PRTN3** | 6,417 | 6,373 | 0,835 | 6,563 | 6,436 | 0,477 | -0,044 | -0,127 | 0,769 |
| **PSGL1** | 5,192 | 5,249 | 0,146 | 5,238 | 5,157 | 0,365 | 0,057 | -0,081 | 0,164 |
| **PSPD** | 3,371 | 3,405 | 0,828 | 3,432 | 3,350 | 0,559 | 0,035 | -0,082 | 0,564 |
| **PTX3** | 4,314 | 4,352 | 0,781 | 4,373 | 4,403 | 0,818 | 0,037 | 0,031 | 0,975 |
| **RAGE** | 13,669 | 13,743 | 0,368 | 13,729 | 13,495 | 0,141 | 0,075 | -0,234 | 0,135 |
| **RARRES2** | 12,182 | 12,281 | 0,335 | 12,259 | 12,226 | 0,828 | 0,099 | -0,033 | 0,454 |
| **REN** | 7,440 | 7,671 | 0,121 | 7,524 | 7,635 | 0,606 | 0,232 | 0,111 | 0,654 |
| **RETN** | 7,163 | 7,183 | 0,906 | 7,305 | 7,231 | 0,676 | 0,019 | -0,074 | 0,702 |
| **SCF** | 10,189 | 10,303 | 0,176 | 10,216 | 10,116 | 0,598 | 0,114 | -0,100 | 0,330 |
| **SCGB3A2** | 2,904 | 3,169 | 0,071 | 3,091 | 3,096 | 0,973 | 0,266 | 0,005 | 0,249 |
| **SELE** | 11,002 | 11,058 | 0,629 | 11,229 | 10,895 | **0,041** | 0,056 | -0,334 | 0,069 |
| **SELP** | 10,906 | 10,687 | 0,382 | 10,777 | 10,711 | 0,638 | -0,220 | -0,066 | 0,606 |
| **SERPINA12** | 3,781 | 4,107 | **0,013** | 3,832 | 3,920 | 0,629 | 0,326 | 0,089 | 0,200 |
| **SHPS1** | 4,200 | 4,341 | 0,164 | 4,372 | 4,152 | 0,175 | 0,141 | -0,220 | **0,032** |
| **SLAMF7** | 4,267 | 4,427 | 0,172 | 4,379 | 4,090 | **0,040** | 0,160 | -0,289 | **0,048** |
| **SOD2** | 10,987 | 11,029 | 0,152 | 11,008 | 10,847 | 0,124 | 0,042 | -0,160 | 0,067 |
| **SORT1** | 10,157 | 10,247 | 0,105 | 10,207 | 10,039 | 0,270 | 0,091 | -0,168 | 0,136 |
| **SPON1** | 1,801 | 1,879 | 0,357 | 1,886 | 1,864 | 0,834 | 0,077 | -0,021 | 0,383 |
| **SPON2** | 9,399 | 9,462 | 0,145 | 9,433 | 9,340 | 0,337 | 0,063 | -0,094 | 0,165 |
| **SRC** | 6,745 | 6,551 | 0,248 | 6,670 | 6,515 | 0,492 | -0,194 | -0,155 | 0,904 |
| **ST2** | 6,936 | 7,102 | 0,133 | 7,161 | 6,912 | 0,128 | 0,166 | -0,249 | **0,030** |
| **STK4** | 2,424 | 2,188 | 0,259 | 2,534 | 2,291 | 0,219 | -0,236 | -0,243 | 0,983 |
| **TF** | 6,436 | 6,520 | 0,337 | 6,483 | 6,282 | 0,225 | 0,085 | -0,201 | 0,086 |
| **TFF3** | 6,386 | 6,405 | 0,873 | 6,481 | 6,434 | 0,711 | 0,019 | -0,048 | 0,705 |
| **TFPI** | 9,506 | 9,446 | 0,782 | 9,476 | 9,430 | 0,844 | -0,060 | -0,046 | 0,964 |
| **TGM2** | 5,944 | 6,180 | 0,093 | 6,366 | 5,865 | 0,120 | 0,236 | -0,501 | **0,048** |
| **THBS2** | 6,504 | 6,570 | 0,178 | 6,533 | 6,439 | 0,311 | 0,067 | -0,095 | 0,135 |
| **THPO** | 4,823 | 4,914 | 0,454 | 4,951 | 4,810 | 0,398 | 0,091 | -0,141 | 0,330 |
| **TIE2** | 7,956 | 8,107 | **0,046** | 8,048 | 7,848 | 0,203 | 0,152 | -0,200 | 0,081 |
| **TIMP4** | 4,559 | 4,752 | 0,189 | 4,730 | 4,574 | 0,417 | 0,192 | -0,156 | 0,118 |
| **TLT2** | 5,762 | 5,902 | 0,334 | 5,959 | 5,773 | 0,296 | 0,139 | -0,186 | 0,139 |
| **TM** | 10,243 | 10,401 | 0,110 | 10,351 | 10,123 | 0,271 | 0,157 | -0,227 | 0,127 |
| **TNFR1** | 7,021 | 7,151 | 0,263 | 7,195 | 7,000 | 0,233 | 0,131 | -0,196 | 0,098 |
| **TNFR2** | 5,837 | 5,979 | 0,256 | 5,987 | 5,786 | 0,241 | 0,141 | -0,201 | 0,098 |
| **TNFRSF10A** | 3,826 | 4,004 | **0,035** | 3,981 | 3,820 | 0,236 | 0,178 | -0,161 | **0,043** |
| **TNFRSF10C** | 5,100 | 5,200 | 0,396 | 5,334 | 5,049 | 0,070 | 0,100 | -0,285 | **0,039** |
| **TNFRSF11A** | 6,721 | 6,875 | 0,112 | 6,791 | 6,643 | 0,389 | 0,155 | -0,148 | 0,175 |
| **TNFRSF13B** | 10,360 | 10,486 | 0,119 | 10,407 | 10,287 | 0,462 | 0,126 | -0,120 | 0,215 |
| **TNFRSF14** | 5,953 | 6,056 | 0,365 | 6,100 | 5,941 | 0,335 | 0,103 | -0,159 | 0,154 |
| **TNFSF13B** | 7,196 | 7,313 | 0,258 | 7,378 | 7,144 | 0,197 | 0,117 | -0,234 | 0,070 |
| **TR** | 5,424 | 5,519 | 0,397 | 5,734 | 5,724 | 0,945 | 0,095 | -0,010 | 0,530 |
| **TRAILR2** | 6,967 | 7,090 | 0,170 | 7,060 | 6,967 | 0,501 | 0,123 | -0,093 | 0,249 |
| **TRAP** | 5,099 | 5,270 | 0,184 | 5,349 | 5,081 | 0,110 | 0,171 | -0,268 | **0,041** |
| **UPAR** | 6,164 | 6,160 | 0,977 | 6,370 | 6,151 | 0,199 | -0,004 | -0,219 | 0,362 |
| **VEGFD** | 8,712 | 8,820 | 0,160 | 8,751 | 8,545 | 0,193 | 0,108 | -0,207 | 0,109 |
| **VSIG2** | 4,976 | 5,247 | **0,006** | 5,105 | 5,028 | 0,611 | 0,271 | -0,077 | 0,113 |
| **XCL1** | 6,219 | 6,339 | 0,279 | 6,340 | 6,174 | 0,309 | 0,120 | -0,166 | 0,206 |
| **hOSCAR** | 11,582 | 11,692 | **0,039** | 11,623 | 11,491 | 0,341 | 0,109 | -0,132 | 0,113 |
| **tPA** | 6,144 | 6,195 | 0,852 | 6,024 | 5,960 | 0,825 | 0,051 | -0,064 | 0,784 |
| **uPA** | 5,764 | 5,810 | 0,754 | 5,829 | 5,706 | 0,435 | 0,046 | -0,123 | 0,426 |
| **vWF** | 7,706 | 7,589 | 0,634 | 7,495 | 7,425 | 0,812 | -0,117 | -0,070 | 0,900 |
