# Supplementary material for: Effect of empagliflozin on coronary microvascular function in patients with type 2 diabetes mellitus–A randomized, placebo-controlled cross-over study
Source: PLoS One. 2022 Feb 11;17(2):e0263481. doi: 10.1371/journal.pone.0263481 (PMC8836314; doi:10.1371/journal.pone.0263481)
Supplement: S1 File — (PDF) [file pone.0263481.s002.pdf]

TITLE	EFFECT OF SGLT2 INHIBITION ON CORONARY MICROVASCULAR FUNCTION IN TYPE 2 DIABETES.
VERSION	3
DATE	10.05.2017
SPONSOR	EVA PRESCOTT, MD DMSC
INVESTIGATORS	METTE ZANDER , MD, PHD<br>MALIN NILSSON, MD<br>HANNAH ELENA SUHRS, MD
CONTACT INFORMATION	HANNAH ELENA SUHRS, MD<br>BISPEBJERG HOSPITAL<br>BISPEBJERG BAKKE<br>2300 COPENHAGEN<br>DENMARK

THE STUDY WILL BE CONDUCTED IN ACCORDANCE WITH THIS PROTOCOL, THE GUIDELINES FOR GOOD CLINICAL PRACTICE AND CURRENT REGULATIONS BY THE AUTHORITIES.

TABLE OF CONTENTS

ABBREVIATIONS **3**
BACKGROUND **3**
OBJECTIVE **5**
HYPOTHESIS **5**
PERSPECTIVE **5**
PLAN AND METHODOLOGY **5**
STUDY POPULATION 5
STUDY DESIGN 5
RANDOMIZATION PROCEDURE 5
FIGURE 1: SPIN OFF THE STUDY 6
TRIAL MEDICINE AND BLINDING 8
SOURCE DATA 9
RESEARCH SUBJECTS **10**
INCLUSION CRITERIA 10
EXCLUSION CRITERIA 10
WITHDRAWAL CRITERIA 10
ENDPOINTS **11**
OUTCOME MEASURES AND THEIR JUSTIFICATION **11**
ECHOCARDIOGRAPHY -CFVR 11
ECHOCARDIOGRAPHY -STRAIN 12
PARACLINICAL DATA 12
DESCRIPTION OF DIAGNOSTIC METHODS **12**
ECHOCARDIOGRAPHY 12
PARACLINICAL DATA 12
BIOBANK **12**
STATISTICS **13**
TIME SCHEDULE **13**
RISKS AND SIDE EFFECTS **13**
MEDICATION 13
CORONARY FLOW VELOCITY RESERVE 14
BLOOD SAMPLES 14
SAFETY **14**
ACCESS TO DATA **15**
QUALITY CONTROL **15**
ETHICAL ASPECTS **15**
INFORMED CONSENT 16
DATA STORAGE **16**
FINANCES **17**
PUBLICATION **17**
REFERENCES **17**

ABBREVIATIONS

CAD	coronary artery disease
CFVR	coronary flow velocity reserve
LAD	left anterior descending artery
SGLT2	sodium-glucose cotransporter 2
SUSAR	suspected unexpected serious adverse reactions
TTDSE	transthoracic Doppler stress echocardiography

BACKGROUND

Type 2 diabetes with concomitant cardiovascular disease increases the risk of death substantially.(1) The EMPA-REG OUTCOME trial investigated the effect of empagliflozin, an inhibitor of the sodium-glucose cotransporter 2 (SGLT2), on cardiovascular morbidity and mortality in patients with type 2 diabetes at high risk of cardiovascular events.(2) They found that those who received empagliflozin had significantly lower rates of the primary composite endpoint of death from cardiovascular causes, nonfatal myocardial infarction or nonfatal stroke than did those who received placebo. The difference in death was driven by a significant reduction in death from cardiovascular causes with a relative risk reduction of 38%. Further, empagliflozin reduced heart failure hospitalization with a relative risk reduction of 35% and a similar benefit in patients with and without baseline heart failure.(3) The cardiovascular effects, safety and efficacy are currently being investigated specifically in heart failure patients with type 2 diabetes in the REFORM trial using cardiac magnetic resonance imaging.(4)

Inhibitors of the SGLT2 reduce rates of hyperglycemia in patients with type 2 diabetes by decreasing renal glucose reabsorption, thereby increasing urinary glucose excretion.(5) Empagliflozin is a selective inhibitor of SGLT2 that has been approved for treatment of type 2 diabetes.(6) Given as either monotherapy or as an add-on therapy, the drug is reported to reduce glycated hemoglobin levels in patients with type 2 diabetes, including those with stage 2 or 3a chronic kidney disease.(7) Empagliflozin is associated with improvement in cardiovascular risk factors. Reduction in systolic blood pressure in the magnitude of 3-4 mmHg and diastolic blood pressure of 1-2 mmHg without increases in heart rate have been reported. (8) Further, empagliflozin has favorable effects on markers of arterial stiffness and vascular resistance (9) as well as albuminuria, (7) and plasma urate. (10-15) Some

effects on plasma lipids are seen. Empagliflozin has been associated with an increase in levels of both low-density lipoprotein and high-density lipoprotein cholesterol. (10-13) Weight reductions of 1.5-2.5 kg over 24-52 weeks have been observed. (13, 14)The additional effect of increasing dose from 10 mg to 25 mg on body weight is modest. Body composition studies assessing changes in visceral adiposity by dual-energy X-ray absorptiometry, computer tomography imaging or magnetic resonance imaging have demonstrated that the weight loss associated with SGLT2 inhibition was due to reduction in visceral or subcutaneous fat. (16-18)

The mechanisms through which SGLT2 inhibition improves cardiovascular prognosis are not known. In the EMPA-REG Outcomes trial the cardiovascular benefits were already seen within 3 months, leading to speculations that osmotic diuresis may be responsible, since other mechanisms like left ventricular remodelling and atherosclerosis would take longer to manifest.(19) It has also been hypothesized that a shift in substrate selection from fatty acid to betahydroxybutyrate oxidation in the heart improves the transduction of oxygen consumtion into work efficiency at the mitochondrial level. Treatment with SGLT2-inhibitor induces mild, persisting hyperketonemia and betahydroxybutyrate is taken up by the heart and oxidized in preference to fatty acids. This mechanism may contribute to the cardioprotection seen in theEMPA-REG OUTCOME trial. Another possible explanation is that the beneficial effects on cardiovascular outcomes are caused by improvement in coronary microvascular function. Coronary microvascular function is impaired in patients with diabetes and heart failure and is strongly associated with poorer cardiovascular outcomes. Coronary microvascular dysfunction has been shown to improve with various interventions, including weight loss, and may contribute to explaining the improved prognosis following intervention.

Coronary microvascular function can be assessed by transthoracic Doppler stress echocardiography (TTDSE) measuring the coronary flow velocity reserve (CFVR) with good reproducibility. (20) CFVR has proven to be a strong prognostic marker of poor cardiovascular prognosis in patients with suspected coronary artery disease.(21-23) In patients with type 2 diabetes and no history of CAD studies have shown that patients with a low CFVR had a significantly higher event rate of cardiovascular endpoints compared to patients with higher CFVR. (24, 25) Diabetes mellitus type 2 has been associated with reduced CFVR (26) and therefore interventions that improve microvascular function in these patients are of great interest.

OBJECTIVE

The aim of the present study is to evaluate the effect of treatment with SGLT2 inhibitors on the coronary microvasculature in patients with type 2 diabetes mellitus.

HYPOTHESIS

We hypothesize that SGLT2 inhibition improves cardiac function through effects on metabolism and coronary microvascular function.

PERSPECTIVE

Knowledge of the mechanisms behind the effects of SGLT2- inhibition could result in an adjustment in guidelines for diabetic care leading to the use of SGLT2 inhibitors as first-line treatment choice in specific subpopulations of patients with diabetes type 2.

PLAN AND METHODOLOGY

STUDY POPULATION

Study participants are recruited from the diabetes out-patient clinic at Bispebjerg University Hospital. Further, patients with diabetes mellitus attending cardiac rehabilitation or the cardiac ambulatory at Bispebjerg University Hospital, and patients with diabetes mellitus attending the iPower study (chest pain and no obstructive coronary stenosis) will be invited to participate.

STUDY DESIGN

Randomized, double-blind, placebo controlled cross-over study of Jardiance 25 mg for 12 weeks and placebo for 12 weeks.

RANDOMIZATION PROCEDURE

Twenty six patients will be randomized to sequence A or B receiving (see figure 1) Empagliflozin and placebo for 12 weeks each in a random order. Randomization is done by the pharmacy (Glostrup Apotek). Id-numbers (1-26) are allocated equally to each treatment sequence using simple randomization. Id-numbers (1-26) will be allocated to patients in a consecutive order.
Randomization is concealed in a document kept by the sponsor, and can only be revealed at the end of the study. For individual patients in occurrence of a serious adverse event the sponsor or investigator will contact the pharmacy to reveal

medication type. The sponsor, investigators and patients will have no knowledge of which sequence the patients belong to.

FIGURE 1: STUDY PROGRESSION

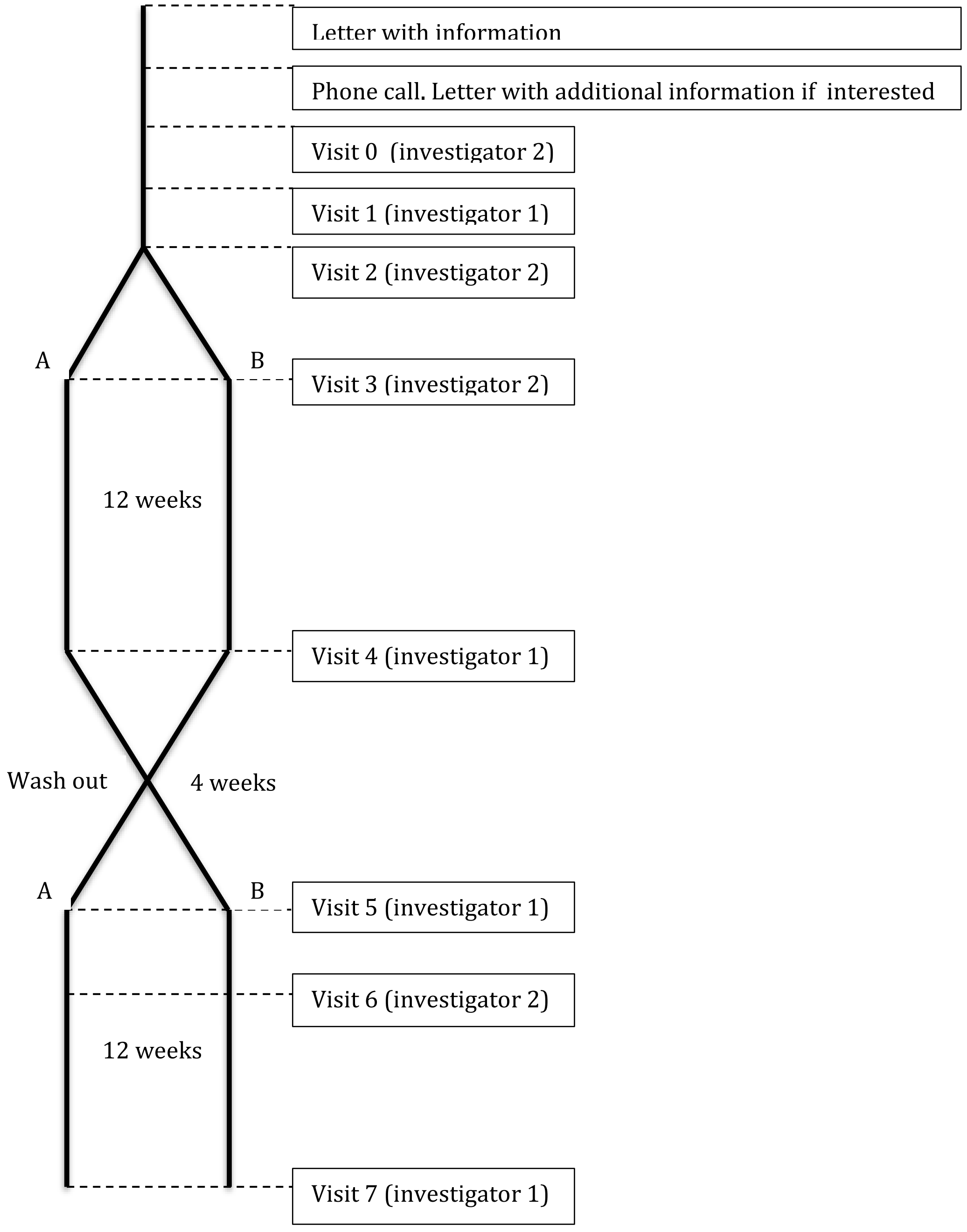

Visit 0 (day-14-0): -informed consent

-blood samples (Hemoglobin, Hba1c, creatine, eGFR)

- blood hcg (fertile women)

Visit 1 (day 0):

- measurement of weight, abdominal circumference and blood pressure
- blood samples (betahydroxybutyrat, Acetoacetat, Insulin, C-peptide, Free Fatty Acids)
- TTDSE incl. CFVR

Visit 2 (0-14 days from visit 1):

- randomization
- treatment initialization (1. period)

Visit 3 (7-21 days from visit 2):

- measurement of weight, abdominal circumference and blood pressure
- blood samples (Hemoglobin, Glucose, Hba1c, creatine, eGFR)
- control of side effects
- control of compliance

Visit 4 (77-91 days from visit 2):

- measurement of weight, abdominal circumference and blood pressure
- blood samples (Hemoglobin, Glucose, Hba1c, creatine, eGFR, betahydroxybutyrat, Acetoacetat, Insulin, C-peptide, Free Fatty Acids)
- TTDSE incl. CFVR
- collection of empty pill bottles

Visit 5 (14-28 days from visit 4):

- measurement of weight, abdominal circumference and blood pressure
- blood samples (Hemoglobin, Glucose, Hba1c, creatine, eGFR, betahydroxybutyrat, Acetoacetat, Insulin, C-peptide, Free Fatty Acids)
- TTDSE incl. CFVR
- treatment initialization (2. period)

Visit 6 (7-21 days from visit 5):

- measurement of weight, abdominal circumference and blood pressure
- blood samples (Hemoglobin, Glucose, Hba1c, creatine, eGFR)
- control of side effects
- control of compliance

Visit 7 (77-91 days from visit 5):

- measurement of weight, abdominal circumference and blood pressure
- blood samples (Hemoglobin, Glucose, Hba1c, creatine, eGFR, betahydroxybutyrat, Acetoacetat, Insulin, C-peptide, Free Fatty Acids)
- TTDSE incl. CFVR
- collection of empty pill bottles

Compliance and adverse events will be checked regularly by phone calls in addition to the scheduled visits. Patients will be contacted by phone 24 hours- 1 week after end of treatment to check for adverse events (plasma half-life is approximately 12 hours).

TRIAL MEDICINE AND BLINDING

Study medication will be prepared by the pharmacy (Glostrup Apotek) in a double-blinded manner. Medication will be delivered in bottles containing 30/90 x 25 mg placebo tablets or 25 mg Empagliflozin (Jardiance®, Boehringer Ingelheim) and both bottle containers and tablets are indistinguishable. Bottle containers will be labelled with a study id-number 1-26 (identification number/treatment number), the batch and/or code number to identify the contents and packaging operation, the name of the investigator Malin Nilsson and mobile number, pharmaceutical dosage form, route of administration, quantity of dosage units, indication of use: “clinical trial”, the storage conditions, period of use (expiry date) and warning label: “keep out of reach of children”.

Blood pressure and kidney function will be controlled at certain time-points (see fig. 1). If oral Empagliflozin is not tolerated treatment will be discontinued. Regarding treatment discontinuation please see ”withdrawal criteria”.

To assure blinding of the investigator assessing the primary endpoint measure, “investigator 1” will be in charge of patient recruitment and echocardiographic examinations. After randomization, patients will be monitored by “investigator 2” (see figure 1). In case of any adverse events/reactions or serious events/reactions “investigator 2” will confer with the sponsor or a third investigator if necessary (and not the investigator assessing the primary endpoint to avoid awareness of medication type). Only in case of a suspected serious adverse reaction medication type will be unblinded by the sponsor or investigator by calling the pharmacy, Døgnåbent Glostrup Apotek, with patients id/cpr-number. Regarding exclusion for unblinding please see ”withdrawal criteria”.
Control of compliance will be made at visit 3 and 6. At the end of the study the patient will return used pill containers with remaining tablets within. These will be counted again to ensure medication compliance and accuracy.

SOURCE DATA

Indication of data and where to find the original source data:

- Allocation of id-number: booking calendar /eCRF
- Blood pressure monitoring: directly written in eCFR
- Weight and abdominal circumference: directly written in eCFR
- Biochemistry: hospital electronic system (LABKA)
- In/exclusion criteria check: directly written in eCRF
    a. Type 2 diabetes: prescription of antiglycemic medication in FMK/EPM
    b. eGFR: LABKA
    c. Previous myocardial infarction of LAD supply area: OPUS
    d. Previous coronary artery by-pass graft (CABG) operation: OPUS
    e. Reversible myocardial ischemia: echopac
    f. Moderate-severe liver disease: OPUS
- Adverse events/reactions: directly written in eCRF or patient journal
- Medicine record (pause/compliance): directly written in eCRF
- Echocardiographic measurements: in worksheet of echopac analysis program

RESEARCH SUBJECTS

INCLUSION CRITERIA

- Type 2 diabetes mellitus defined as medical treatment with antidiabetic drugs for 12 weeks prior to randomization
- 40-80 years of age
- eGFR≥45 l per minute per 1.73 m^2 of body surface area

EXCLUSION CRITERIA

- Allergy to Empagliflozin or its components
- Ongoing treatment with SGLT2 inhibitor or treatment within the last four weeks.
- Hba1c<58 mmol/mol
- Previous myocardial infarction involving the LAD supply area
- Previous coronary artery by-pass graft (CABG) operation
- Reversible myocardial ischemia assessed by adenosine stress echocardiography
- Indication of moderate-severe liver disease
- Pre-menopausal women (last menstruation ≤1 year prior to informed consent) who are nursing, pregnant, or of child-bearing potential and are not practicing an acceptable method of birth control throughout the study. Acceptable methods of birth control includehormonal contraceptives consisting of a combination of estrogen and progesterone (the contraceptive pill, ring or rod) or consisting of progesterone only (mini-pill, progesterone injection or implant). Further, hormone or cobber coil may be used. ,
- Pregnancy (pregnancy test will be done at inclusion in fertile women)
- Alcohol or drug abuse within 3 months of informed consent that would interfere with trial participation or any ongoing condition leading to decreased compliance with study procedures or study drug intake
- Intake of an investigational drug in another trial within 30 days prior to intake of study medication in this trial or participating in another trial involving an investigational drug.

WITHDRAWAL CRITERIA

- Sudden unexpected serious adverse reaction
- If the subject does not want to continue treatment
- Sustained side effects that make the patient unable to continue treatment

- GFR < 30 mL/min/1,73 m2 and/or >20 % decrease in eGFR during commencement of treatment
- If insulin treatment is reduced more than 20% due to hypoglycaemia.
- Patients who do not wish any endpoint measures
- Compliance less than 70%

Study subjects who withdraw due to a sudden unexpected serious adverse reaction, a serious adverse reaction or sustained adverse reaction will be followed throughout the study period.
Patients excluded due to poor compliance or if they do not wish to participate in outcome measurements will no longer be followed but will need to seek their general practitioner regarding any health problem as they normally would.

ENDPOINTS

Primary

- Changes in CFVR assessed by TTDSE.

Secondary

- Changes in cardiac function assessed by speckle tracking echocardiography
- Changes in biochemical markers of cardiac metabolism

OUTCOME MEASURES AND THEIR JUSTIFICATION

ECHOCARDIOGRAPHY -CFVR

Coronary flow velocity reserve is a measure of microvessel dysfunction in the absence of upstream coronary stenosis. CFVR will be measured with transthoracic doppler stress echocardiography of the left anterior descending artery (LAD) before and during infusion of adenosine. The feasibility of CFVR measurement by Doppler echocardiography has been assessed in the iPower population (angina and no macrovascular coronary stenosis). CFVR could be reliably assessed on the LAD with good quality in 97% of the population. (20) CFVR measured by this method has been shown to be a strong risk marker in patients with non-obstructive coronary artery disease. (22)
The repeatability of transthoracic echocardiography CFVR measurement has been evaluated in 10 healthy volunteers (4 female, 6 male) and 10 women with angina and no obstructive coronary artery disease (CAD). Reliability of CFVR measurements was high in healthy volunteers, 97% (CI, 92%; 101%) of the variability was estimated to be due to genuine differences in CFVR and the remaining 3% was estimated to be

due to error in measurements. In the women with angina and no obstructive CAD, the intraclass correlation coefficient (CI) was 90% (78%; 102). The variance in the two populations was similar (p=0.81). A pooled analysis yielded limits of agreement (2xSD) (CI) of 0.45 (0.31;0.61) and an intraclass correlation coefficient (CI) of 96% (92%;99%). (27)

Echocardiography -Strain

Improvement in cardiac function following intervention may be subtle and is better measured during stress. We aim to determine the long term effects of SGLT2 inhibition on cardiac function. By using global longitudinal and radial 2D strain at rest and during adenosine stress, we expect that we will be able to detect a smaller difference in myocardial function than by using change in left ventricular ejection fraction assessed by the Simpsons method.

Paraclinical data

Under conditions of hyperkentonemia, use of betahydroxybutyrate as fuel source improves the transduction of oxygen consumption into work efficiency in the endangered myocardium. This mechanism may coorporate with other changes after SGLT2-inhibitor treatment to achieve the degree of cardioprotection revealed by the EMPA-REG OUTCOME trial.

Description of diagnostic methods

Echocardiography

Echocardiographic measurements of CFVR, systolic and diastolic function including strain will be done both at rest and during stress. CFVR is calculated as the ratio between coronary flow velocity during hyperemia and rest. Coronary microvascular dysfunction is defined as CFVR<2.5. We aim at detecting an improvement in CFVR regardless of the baseline value.

Paraclinical data

Blood samples (approximately 10 ml) will be drawn at visit 3 and 6 (Hemoglobin, Glucose, Hba1c, creatine, eGFR) to monitor renal function and blood glucose. Additional fasting blood samples (approximately 15 ml) will be drawn at visit 1, 4, 5 and 7 (betahydroxybutyrat, Acetoacetat, Insulin, C-peptide, Free Fatty Acids) to monitor sugar and lipid metabolism.

Biobank

Some of the blood samples (betahydroxybutyrat, Acetoacetat, Insulin, C-peptide, Free Fatty Acids) may be analysed at the end of the study, thus a new project biobank will be created for the project for storage of blood samples. Blood samples will be stored until the end of the study (last patient, last visit) plus five years, and they will be destroyed thereafter. The other blood samples (Hemoglobin, Glucose, Hba1c, creatine, eGFR) will be analysed immediately and destroyed thereafter.

STATISTICS

CFVR is a continuous response variable. We hypothesized that the SGLT2-inhibitor treated group would have a higher CFVR at 12 weeks compared to placebo. A reproducibility study in iPower women (angina-like chest pain and no coronary stenosis) at our clinic with same echocardiographer for the present study had a SD of 0.24 on repeated measurements within 10 days. Furthermore a study with diabetes patients had a SD of 0.32 on repeated measurements and 0.38 on repeated measurements after treatment with GLP1-analogue. We therefore assume that the SD on mean difference in CFVR will be 0.35 in our population. An improvement of 0.23 (i.e. approx. 10%) in CFVR is regarded as clinically relevant. An estimated sample size of 21 was calculated to be necessary for detection of a 0.23 difference in paired means of CFVR with a power of 80% and a two –sided significance level of 5%. Anticipating a 20% dropout rate, enrolment was set at 26 patients.

TIME SCHEDULE

April 2017 – July 2017: inclusion and randomization
April 2017 – Januar 2018: Intervention and finalization
February 2018 – March 2018: Data analysis, article writing and publication

RISKS AND SIDE EFFECTS

MEDICATION

The most frequent side effect is hypoglycaemia when combined with sulfonylurea or insulin. It is common to experience genital infection or urinary tract infections due to the increased glucoseuria. Likewise pruritus and increased urination is common. It is uncommon to experience volume depletion or decrease in renal function, and diabetic ketoacidosis is very rare.
Insulin-treated patients may need reduction in insulin treatment to avoid risk of hypoglycaemia. They will continue to measure blood glucose at least two times a day as it is normally recommended and a diary of blood sugar measurements will be

handed out for them to complete. Every 14th day they will be contacted by phone by a study investigator (not doing endpoint measurements) and based on their registered blood sugars they will be advised to continue or adjust their insulin dosage. Patients on insulin treatment will be informed to contact study investigator if their blood sugar is <5 mmol/L on a measurement.
Given the possible advantage of better glycaemic control and improvement in cardiovascular risk factors we consider the risks to be offset by the advantages. All patients will be informed about side effects. If a patient develops sustained or severe side effects he/she will be told to discontinue study treatment.

Coronary flow velocity reserve

Adenosine is routinely used in clinical practice for SPECT scans. Administration of adenosine has several potential side effects. The most common are shortness of breath, drop in blood pressure, flushing, headache, bradycardia and 3rd degree AV blockage (www.medicin.dk). These side effects are generally mild and of short duration because of the very short half time of adenosine (seconds). All side effects are typically remitted approximately 1 minute after infusion has stopped. If the patient experiences more pronounced side effects the infusion is discontinued. Advanced resuscitation equipment will always be accessible when examination is conducted.

Blood samples

All blood samples will be taken under sterile conditions and the risk of infection is minimal. Some discomfort may be related to the needle.
After examination procedures the patients will be observed in the clinic for 30 minutes before they are allowed to leave the hospital.

Safety

Sponsor is responsible that all information on suspected unexpected serious adverse reactions (SUSAR) that may be fatal or life threatening are registered and informed to Lægemiddelstyrelsen as soon as possible, and within seven days after sponsor is informed about the event. Within eight days after reporting the event it is the responsibility of the sponsor to pass all relevant information about the follow up on the reporting to Lægemiddelstyrelsen.
All other unexpected and serious suspected adverse reactions will be reported to Lægemiddelstyrelsen within 15 days after sponsor is informed about the event. Every report will have a comment on any consequences that it may have for the study.
Serious adverse reactions or events, not considered SUSAR are registered in the

eCRF and reported 1 time yearly to Lægemiddelstyrelsen and Videnskabsetisk Komite.
A serious adverse event or a serious adverse reaction is defined as an event or reaction that no matter dose will result in death, be life-threatening, lead to hospital admission or extension of hospital admission, result in significant or sustained invalidity, unability to work or will lead to congenital anomalies or malformations.
An adverse event is any undesirable event experienced by a study participant in a clinical trial after treatment with a study drug, regardless that there may not be any causality between the study drug and the event.
An adverse reaction is any undesirable reaction to a study drug no matter the dose.
Investigator will evaluate the possible causality of an event. Investigator will register all adverse reactions and adverse events (including those categorized as serious), and/or abnormal results that may represent a risk for study participants. Investigator will immediately report all serious adverse events to sponsor. Study participants have to be identified by a personal identification number.
Sponsor will produce a list of all serious suspected adverse reactions that have occurred in the investigation period and a report on the safety of the study participants. The list and report is sent to Lægemiddelstyrelsen.
Registries will take place from the time of inclusion until the last examination has taken place.
The documents of reference safety information (RSI) are the European Public Assessment Report (EPAR) Summery of product characteristics and the package leaflet.
Patients who experience SUSAR or any other suspected serious adverse reaction will be followed until the last visit. Hereafter the patient will be followed by the general practitioner.

ACCESS TO DATA

Lægemiddelstyrelsen, the GCP unit, Videnskabsetisk komite and Datatilsynet will be given direct access to data by the investigator.

QUALITY CONTROL

Procedures for quality control will be established. The GCP unit at Bispebjerg Hospital will be responsible for monitoring the study. Audits and inspections will be allowed.

ETHICAL ASPECTS

Only patients who give informed consent will participate in the study. Information about study participants will be protected according to ‘lov om behandling af personoplysninger’ and ‘Sundhedsloven’. Patients will be informed about compensation schemes according to ‘lov om klage og erstatningsadgang inden for sundhedsvæsenet’. The study is sent to Lægemiddelstyrelsen, Datatilsynet and Videnskabsetisk Komite.
Patients can contact medical doctor Malin Nilsson at any time for information regarding the project.

Patients will receive study medication according to clinical guidelines. They will be randomized to SGLT2 inhibition or placebo in addition to their usual diabetic medication. Randomization will be performed by the pharmacy and health care providers and patients will be blinded to treatment allocation.
According to previous trials described above, patients with diabetes mellitus are at increased risk of cardiovascular disease. This study will describe the possible mechanisms behind the effect of treatment with SGLT2-inhibitors on cardiac function, and it may add to the understanding of microvessel disease. Understanding of the mechanisms behind the cardiovascular benefits of SGLT2 inhibition may guide the choice of antidiabetic drug in different patient subgroups with diabetes in the future.

Informed consent

Patients are recruited on the basis of in- and exclusion criteria. They will receive a letter with information about the study, and subsequently they will be contacted by phone, and if they are interested, oral information about the project will be given by a member of the study group. If they have interest in participating in the project a meeting will be arranged and they will be informed that they can bring an assessor. A letter with additional information and confirmation of the appointment date and time will be sent. Further information will be given at their first appointment in an undisturbed room by one of the investigators. If the patient consents, the form is signed (“Samtykkeerklæring”) and the patient receives a copy of the informed consent.

Data storage

Every patient will receive an individual study number. All data on every patient will be registered under this number.
All data will be stored securely in a locked office and in an electronic CRF for five years.

FINANCES

Professor Eva Prescott, Department of Cardiology, Bispebjerg University Hospital is sponsor of the present project. The project is financed partly by "Forsknings- og Innovationsstyrelsen" (from a phd-grant of approximately 1.5 millions). No reimbursement is given to the study subjects for participating in this project.

We will continue to apply for non-commercial funds. In the case of any new grants for the project the "Videnskabsetiske Komiteer" and study participants will be informed about the amount and who has given the grant.

The sponsor or investigators have no commercial interests in this project.

PUBLICATION

Both positive, negative and inconclusive data from this study will be published in medical journals and as abstracts at congresses. The study will be registered on clinicaltrials.gov.

REFERENCES

1. Emerging Risk Factors C, Di Angelantonio E, Kaptoge S, Wormser D, Willeit P, Butterworth AS, et al. Association of Cardiometabolic Multimorbidity With Mortality. JAMA. 2015;314(1):52-60.
2. Zinman B, Wanner C, Lachin JM, Fitchett D, Bluhmki E, Hantel S, et al. Empagliflozin, Cardiovascular Outcomes, and Mortality in Type 2 Diabetes. N Engl J Med. 2015;373(22):2117-28.
3. Fitchett D, Zinman B, Wanner C, Lachin JM, Hantel S, Salsali A, et al. Heart failure outcomes with empagliflozin in patients with type 2 diabetes at high cardiovascular risk: results of the EMPA-REG OUTCOME(R) trial. Eur Heart J. 2016;37(19):1526-34.
4. Singh JS, Fathi A, Vickneson K, Mordi I, Mohan M, Houston JG, et al. Research into the effect Of SGLT2 inhibition on left ventricular remodelling in patients with heart failure and diabetes mellitus (REFORM) trial rationale and design. Cardiovasc Diabetol. 2016;15:97.
5. Gallo LA, Wright EM, Vallon V. Probing SGLT2 as a therapeutic target for diabetes: basic physiology and consequences. Diab Vasc Dis Res. 2015;12(2):78-89.
6. Grempler R, Thomas L, Eckhardt M, Himmelsbach F, Sauer A, Sharp DE, et al. Empagliflozin, a novel selective sodium glucose cotransporter-2 (SGLT-2) inhibitor: characterisation and comparison with other SGLT-2 inhibitors. Diabetes Obes Metab. 2012;14(1):83-90.
7. Barnett AH, Mithal A, Manassie J, Jones R, Rattunde H, Woerle HJ, et al. Efficacy and safety of empagliflozin added to existing antidiabetes treatment in patients with type 2 diabetes and chronic kidney disease: a randomised, double-

blind, placebo-controlled trial. The Lancet Diabetes & Endocrinology. 2014;2(5):369-84.
8. Tikkanen I, Narko K, Zeller C, Green A, Salsali A, Broedl UC, et al. Empagliflozin reduces blood pressure in patients with type 2 diabetes and hypertension. Diabetes Care. 2015;38(3):420-8.
9. Chilton R, Tikkanen I, Cannon CP, Crowe S, Woerle HJ, Broedl UC, et al. Effects of empagliflozin on blood pressure and markers of arterial stiffness and vascular resistance in patients with type 2 diabetes. Diabetes Obes Metab. 2015;17(12):1180-93.
10. Haring HU, Merker L, Seewaldt-Becker E, Weimer M, Meinicke T, Broedl UC, et al. Empagliflozin as add-on to metformin in patients with type 2 diabetes: a 24-week, randomized, double-blind, placebo-controlled trial. Diabetes Care. 2014;37(6):1650-9.
11. Haring HU, Merker L, Seewaldt-Becker E, Weimer M, Meinicke T, Woerle HJ, et al. Empagliflozin as add-on to metformin plus sulfonylurea in patients with type 2 diabetes: a 24-week, randomized, double-blind, placebo-controlled trial. Diabetes Care. 2013;36(11):3396-404.
12. Roden M, Weng J, Eilbracht J, Delafont B, Kim G, Woerle HJ, et al. Empagliflozin monotherapy with sitagliptin as an active comparator in patients with type 2 diabetes: a randomised, double-blind, placebo-controlled, phase 3 trial. The Lancet Diabetes & Endocrinology. 2013;1(3):208-19.
13. Kovacs CS, Seshiah V, Swallow R, Jones R, Rattunde H, Woerle HJ, et al. Empagliflozin improves glycaemic and weight control as add-on therapy to pioglitazone or pioglitazone plus metformin in patients with type 2 diabetes: a 24-week, randomized, placebo-controlled trial. Diabetes Obes Metab. 2014;16(2):147-58.
14. Rosenstock J, Jelaska A, Frappin G, Salsali A, Kim G, Woerle HJ, et al. Improved glucose control with weight loss, lower insulin doses, and no increased hypoglycemia with empagliflozin added to titrated multiple daily injections of insulin in obese inadequately controlled type 2 diabetes. Diabetes Care. 2014;37(7):1815-23.
15. Rosenstock J, Jelaska A, Zeller C, Kim G, Broedl UC, Woerle HJ, et al. Impact of empagliflozin added on to basal insulin in type 2 diabetes inadequately controlled on basal insulin: a 78-week randomized, double-blind, placebo-controlled trial. Diabetes Obes Metab. 2015;17(10):936-48.
16. Cefalu WT, Leiter LA, Yoon K-H, Arias P, Niskanen L, Xie J, et al. Efficacy and safety of canagliflozin versus glimepiride in patients with type 2 diabetes inadequately controlled with metformin (CANTATA-SU): 52 week results from a randomised, double-blind, phase 3 non-inferiority trial. The Lancet. 2013;382(9896):941-50.
17. Bolinder J, Ljunggren O, Johansson L, Wilding J, Langkilde AM, Sjostrom CD, et al. Dapagliflozin maintains glycaemic control while reducing weight and body fat mass over 2 years in patients with type 2 diabetes mellitus inadequately controlled on metformin. Diabetes Obes Metab. 2014;16(2):159-69.
18. Ridderstråle M, Andersen KR, Zeller C, Kim G, Woerle HJ, Broedl UC. Comparison of empagliflozin and glimepiride as add-on to metformin in patients with type 2 diabetes: a 104-week randomised, active-controlled, double-blind, phase 3 trial. The Lancet Diabetes & Endocrinology. 2014;2(9):691-700.

19. Inzucchi SE, Zinman B, Wanner C, Ferrari R, Fitchett D, Hantel S, et al. SGLT-2 inhibitors and cardiovascular risk: proposed pathways and review of ongoing outcome trials. Diab Vasc Dis Res. 2015;12(2):90-100.
20. Michelsen MM, Pena A, Mygind ND, Frestad D, Gustafsson I, Hansen HS, et al. Coronary Flow Velocity Reserve Assessed by Transthoracic Doppler: The iPOWER Study: Factors Influencing Feasibility and Quality. J Am Soc Echocardiogr. 2016;29(7):709-16.
21. Rigo F, Cortigiani L, Pasanisi E, Richieri M, Cutaia V, Celestre M, et al. The additional prognostic value of coronary flow reserve on left anterior descending artery in patients with negative stress echo by wall motion criteria. A Transthoracic Vasodilator Stress Echocardiography Study. Am Heart J. 2006;151(1):124-30.
22. Pepine CJ, Anderson RD, Sharaf BL, Reis SE, Smith KM, Handberg EM, et al. Coronary microvascular reactivity to adenosine predicts adverse outcome in women evaluated for suspected ischemia results from the National Heart, Lung and Blood Institute WISE (Women's Ischemia Syndrome Evaluation) study. J Am Coll Cardiol. 2010;55(25):2825-32.
23. Cortigiani L, Rigo F, Gherardi S, Bovenzi F, Molinaro S, Picano E, et al. Coronary flow reserve during dipyridamole stress echocardiography predicts mortality. JACC Cardiovasc Imaging. 2012;5(11):1079-85.
24. Cortigiani L, Rigo F, Gherardi S, Galderisi M, Bovenzi F, Sicari R. Prognostic meaning of coronary microvascular disease in type 2 diabetes mellitus: a transthoracic Doppler echocardiographic study. J Am Soc Echocardiogr. 2014;27(7):742-8.
25. Kawata T, Daimon M, Hasegawa R, Toyoda T, Sekine T, Himi T, et al. Prognostic value of coronary flow reserve assessed by transthoracic Doppler echocardiography on long-term outcome in asymptomatic patients with type 2 diabetes without overt coronary artery disease. Cardiovasc Diabetol. 2013;12:121.
26. Tuccillo B, Accadia M, Rumolo S, Iengo R, D'Andrea A, Granata G, et al. Factors predicting coronary flow reserve impairment in patients evaluated for chest pain: an ultrasound study. J Cardiovasc Med (Hagerstown). 2008;9(3):251-5.
27. Michelsen MM, Mygind ND, Pena A, Olsen RH, Christensen TE, Ghotbi AA, et al. Transthoracic Doppler echocardiography compared with positron emission tomography for assessment of coronary microvascular dysfunction: The iPOWER study. Int J Cardiol. 2016;228:435-43.
